# Enhancing venetoclax efficacy in leukemia through association with HDAC inhibitors

**DOI:** 10.1038/s41420-025-02446-4

**Published:** 2025-04-06

**Authors:** Jorge Antonio Elias Godoy Carlos, Mauricio Temotheo Tavares, Keli Lima, Larissa Costa de Almeida, Karoline de Barros Waitman, Leticia Veras Costa-Lotufo, Roberto Parise-Filho, João Agostinho Machado-Neto

**Affiliations:** 1 Department of Pharmacology, Institute of Biomedical Sciences, University of São Paulo, São Paulo, Brazil; 2 Department of Pharmacy, Faculty of Pharmaceutical Science, University of São Paulo, São Paulo, Brazil; 3 Department of Cancer Biology, Dana-Farber Cancer Institute, Boston, MA 02115 USA; 4 Department of Biological Chemistry and Molecular Pharmacology, Harvard Medical School, Boston, MA 02115 USA; 5 Laboratory of Medical Investigation in Pathogenesis and Targeted Therapy in Onco-Immuno-Hematology (LIM-31), Department of Internal Medicine, Hematology Division, Faculty of Medicine, University of São Paulo, São Paulo, Brazil

**Keywords:** Acute myeloid leukaemia, Pharmacodynamics

## Abstract

Epigenetic modifications significantly influence gene expression and play crucial roles in various biological processes, including carcinogenesis. This study investigates the effects of novel purine-benzohydroxamate compounds, particularly **4** **f**, as hybrid kinase/histone deacetylase (HDAC) inhibitors in hematological malignancies, focusing on acute myeloid leukemia (AML). Our results demonstrate that these compounds selectively reduce cell viability in blood cancer cells, with inhibitory concentration values indicating higher potency against neoplastic cells compared to normal leukocytes. Mechanistically, **4** **f** induces apoptosis and cell cycle arrest, promoting differentiation in leukemia cells, while effectively inhibiting HDAC activity. Furthermore, **4** **f** enhances the therapeutic efficacy of venetoclax, a BCL2 inhibitor, in AML models sensitive and resistant to this drug. The combination treatment significantly increases apoptosis and reduces cell viability, suggesting a synergistic effect that may overcome drug resistance. This study provides valuable insights into the potential of HDAC inhibitors, particularly **4** **f**, as a promising therapeutic strategy for treating resistant hematological malignancies. Our findings underscore the importance of further exploring hybrid kinase/HDAC inhibitors in combination therapies to improve outcomes in patients with acute leukemias and other hematological malignancies.

## Introduction

Epigenetic modifications are heritable changes that modulate the chromatin structure without altering the DNA nucleotide sequence. In humans, epigenetic alterations play a crucial role in regulating gene expression, and various types of these modifications have been extensively documented. The most well-characterized epigenetic modifications include DNA methylation, histone modifications, and non-coding RNAs [1–3]. Among these, the acetylation and deacetylation of histones are post-translational modifications that have been widely studied, playing a key role in chromatin remodeling. This process is regulated by two enzyme families: histone acetyltransferases (HATs) and histone deacetylases (HDACs), which add or remove acetyl groups from the ɛ-amino groups of histone lysine residues, leading to either an open or condensed chromatin structure, thereby promoting gene expression or repression, respectively [4, 5].

The acetylation process mediated by HATs opens the chromatin, allowing the expression of genes involved in processes such as cell cycle progression, apoptosis, and autophagy, which are relevant to carcinogenesis. Conversely, HDACs exert a repressive effect by reducing histone acetylation through the removal of acetyl groups from lysine residues, leading to chromatin condensation and gene repression [6–8]. HDAC inhibitors (HDACi) have been clinically used, either as monotherapy or in combination with other antineoplastic drugs. Numerous clinical trials have investigated various HDACi, offering new perspectives in the treatment of cancer [9, 10].

HDACi have been extensively investigated in hematological malignancies, particularly in acute leukemias, which are diseases with poor prognosis and difficult therapeutic management [11]. In these models, it has been frequently reported that HDAC inhibition leads to histone hyperacetylation, resulting in chromatin decompaction and reactivation of tumor suppressor and differentiation genes [12, 13]. Another important aspect is that, in acute leukemias, HDACi have been mainly studied in combination with other therapies. HDACi monotherapies generally show limited efficacy, but when combined with agents such as DNA methyltransferase inhibitors, targeted therapies, or conventional chemotherapeutics, they demonstrate synergistic potential [14–16]. In this context, the use of HDACi with dual-targeting capabilities may be of interest, as this pharmacological property could offer advantages in overcoming the issues of non-selectivity and drug resistance commonly associated with single-target therapies [17, 18]. Thus, the use of HDACi in acute leukemias is still being refined, focusing on therapeutic combinations and the identification of biomarkers that could predict which patients would benefit most from this type of treatment.

In the present study, we characterized the cellular and molecular effects of novel hybrid kinase/HDAC inhibitors [19], as well as evaluated their combination with venetoclax, a BCL2 inhibitor, in experimental models of acute myeloid leukemia (AML).

## Results

### Purine-benzohydroxamate compounds drive selective reduction of cell viability in cellular models of hematological malignancies

**Fig. 1:**
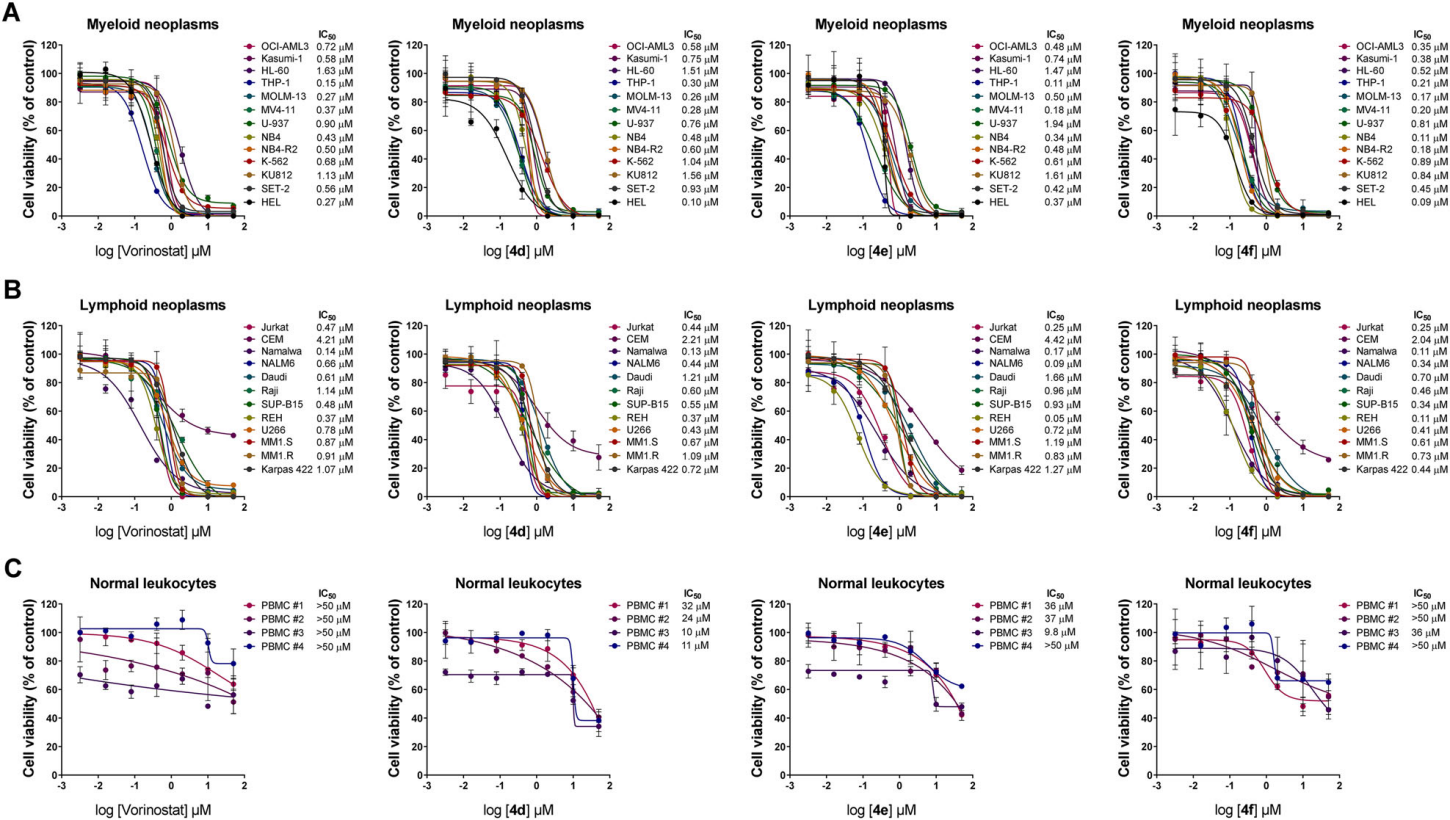
Purine-benzohydroxamate compounds selectively reduce cell viability, with compound 4 f being the most potent in blood cancer models tested. Dose- and time-dependent cytotoxicity was analyzed by methylthiazoletetrazolium (MTT) assay using 13 myeloid neoplasm cell lines (**A**), 12 lymphoid neoplasm cell lines (**B**), and 4 normal leukocyte samples (**C**) treated with vehicle or graded concentrations of vorinostat, **4** **d,** **4e**, or **4** **f** (ranging from 0.0032 to 50 µM) for 72 h. Cell viability was measured as a percentage relative to vehicle-treated controls. Data are presented as mean ± SD from at least three independent experiments. IC_50_ values for each compound and cellular model are described in the Figure.

As previously reported, a series of hybrid inhibitors combining pharmacophores from known kinase inhibitors and HDAC inhibitors were synthesized and initially tested on Jurkat and Namalwa cell lines [19]. In this study, we significantly expand upon these initial findings by evaluating the efficacy of these compounds across a broader panel of hematological malignancies, including cellular models of AML, ALL, MPN, lymphoma, and MM. As shown in Fig. 1A, the compound **4** **d** reduces cell viability with IC_50_ values ranging from 0.10 and 1.56 µM, while **4e** shows IC_50_ values between 0.11 to 1.94 µM, and **4** **f** exhibits IC_50_ values from 0.09 to 0.89 µM in myeloid neoplasm models. In lymphoid neoplasms (Fig. 1B), IC_50_ values range from 0.13 to 2.21 µM for **4** **d**, from 0.05 to 4.42 µM for **4e**, and from 0.11 to 2.04 µM for **4** **f**. Importantly, the compounds demonstrated significantly higher IC_50_ values in normal leukocytes (Fig. 1C), suggesting selective cytotoxicity toward neoplastic cells (selective index ranged from 2.21 to >555-fold). Vorinostat, used as a reference drug, exhibited IC_50_ values ranging from 0.27 to 1.63 µM in myeloid neoplasms, from 0.47 µM to 4.21 µM in lymphoid neoplasms, and >50 µM in normal leukocytes. Given the more pronounced effects observed in myeloid neoplasms, we selected several molecularly distinct models for further investigation. These studies revealed that the reduction induced by the purine-benzohydroxamate compounds in cell viability was both time- and concentration-dependent (*p* < 0.05, Supplementary Fig. 1).

### Purine-benzohydroxamate compounds exhibit multiple antineoplastic effects in leukemia cells

**Fig. 2:**
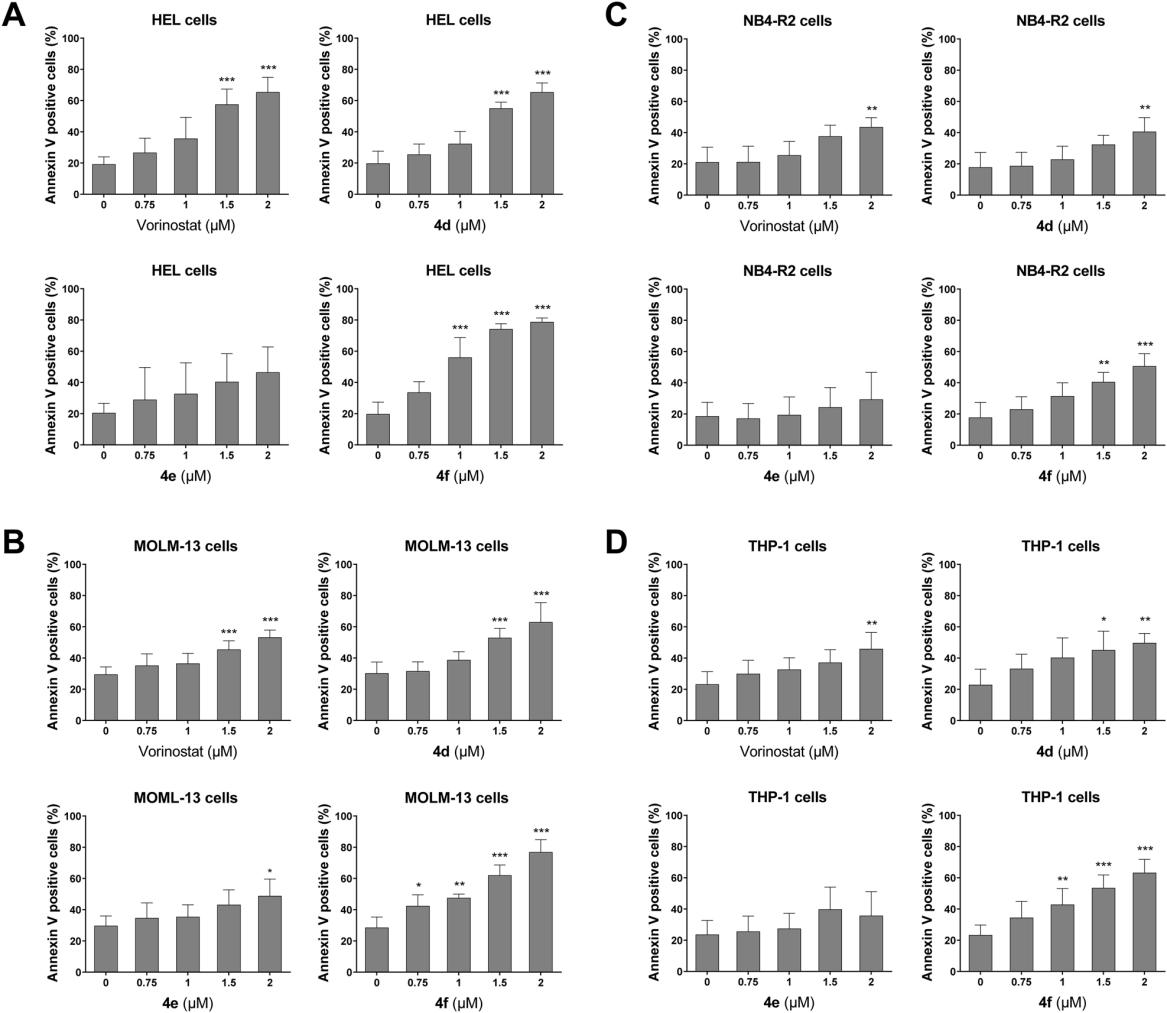
Novel HDAC inhibitors induce apoptosis in acute myeloid leukemia cells. HEL (**A**), MOLM-13 (**B**), NB4-R2 (**C**), and THP-1 (**D**) cells were labeled with APC-annexin V/propidium iodide (PI) after treatment with either vehicle or the indicated concentrations of vorinostat, **4** **d,** **4e**, or **4** **f** for 24 h. Bar graphs show the mean ± SD of at least three independent experiments. The *p* values and cell lines are indicated in the graphs; *p < 0.05, **p < 0.01, ***p < 0.0001; ANOVA and Bonferroni post-test.

**Fig. 3:**
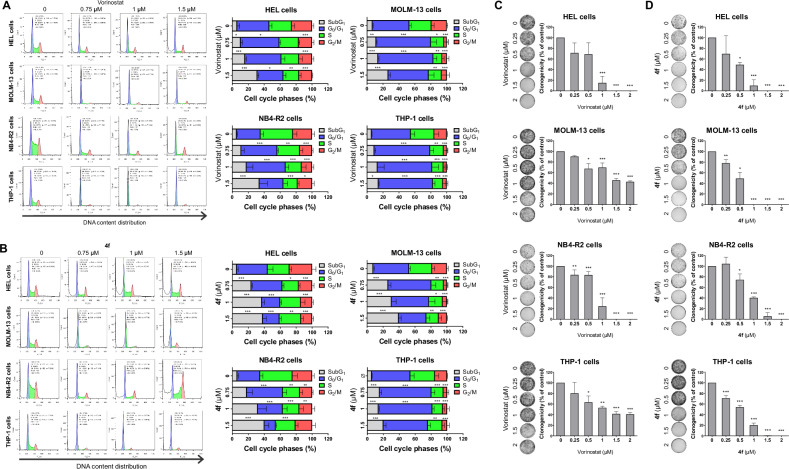
The novel HDAC inhibitor 4 f induces cell cycle arrest and efficiently reduces autonomous clonal growth in acute myeloid leukemia (AML) cells. Cell cycle phases were determined by analyzing DNA content via propidium iodide staining, followed by data acquisition using flow cytometry after treating AML cells with either vehicle, vorinostat (**A**), or **4** **f** (**B**) at the indicated concentrations for 24 h. A representative histogram is shown for each condition. The bar graph displays the mean ± SD of at least three independent experiments. The *p* values and cell lines are indicated in the graphs; *p < 0.05, **p < 0.01, ***p < 0.001; ANOVA and Bonferroni post-test. HEL, MOLM-13, NB4-R2, and THP-1 cells were cultured in a semisolid medium in the presence of either vehicle or increasing concentrations of vorinostat (**C**) or 4 f (**D**). Colonies containing viable cells were detected by adding MTT reagent after 8–12 days of culture. Colony images are shown for a representative experiment, and the bar graphs display the mean ± SD of at least three independent experiments. *p < 0.05, **p < 0.01, ***p < 0.001; ANOVA and Bonferroni *p*ost-test.

Next, potential mechanisms involved in the reduction of cell viability induced by purine-benzohydroxamate compounds were investigated. Firstly, it was observed that the compounds triggered apoptosis, with **4** **f** being the most effective (Fig. 2 and Supplementary Fig. 2). Thus, we continued to deepen the characterization of **4** **f** in the following experiments. At low concentrations, **4** **f** causes a reduction in cell cycle progression with accumulation of cells in the G_0_/G_1_ phases and a reduction in cells in the S, G_2_/M phases. At higher concentrations, there is an accumulation of cells in subG_1_, corroborating the data from the cell death assays (Fig. 3A, B). Autonomous colony formation assays revealed that **4** **f** is more effective than vorinostat, being able to completely inhibit clonogenicity in MOLM-13 and THP-1 cells (Fig. 3C, D).

Given that HDACs contribute as repressors of cell differentiation in AML models [13], we investigated the effects of **4** **f** on this process. In NB4-R2 and THP-1 cells, exposure to **4** **f** led to a significant increase in the cell differentiation marker CD11b, surpassing the effects of vorinostat (Supplementary Fig. 3A). Morphological analysis further revealed that **4** **f** altered the nuclei-to-cytoplasm ratio and increased vacuole formation, which are indicative of a more differentiated cell phenotype (Supplementary Fig. 3b).

### Compound 4 f reduces HDAC activity and favors a tumor suppressive molecular network in AML models

**Fig. 4:**
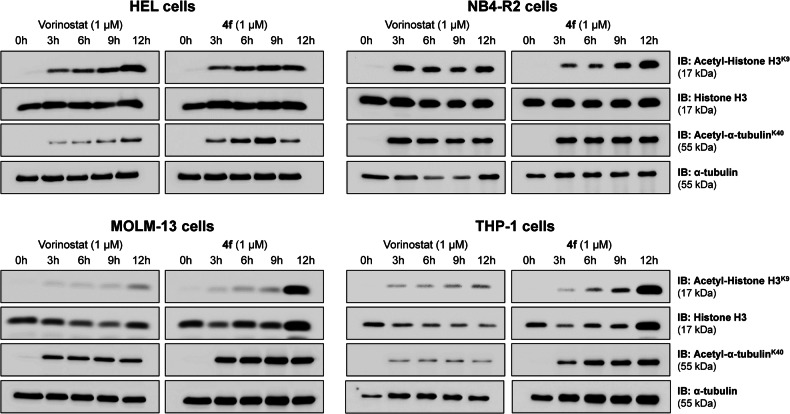
Compound 4 f inhibits HDAC activity in acute myeloid leukemia cells. Western blot analysis was performed to detect acetyl-histone H3 (Lys9), histone H3, acetyl-α-tubulin (Lys40), and α-tubulin in total cell extracts from HEL, NB4-R2, MOLM-13, and THP-1 cells treated with vehicle, vorinostat (1 µM), or **4** **f** (1 µM) for 0, 3, 6, 9, or 12 h. Vorinostat was used as a reference drug. Cell lines are indicated in the Figure.

Exposure to **4** **f** modulated proteins involved in HDAC activity. Specifically, changes were observed in acetyl-α-tubulin (Lys40) and acetyl-histone H3 (Lys9), which are substrates of HDAC6 and HDAC class I, respectively. As shown in Fig. 4, leukemia cells exhibited increased levels of these acetylated proteins, indicating that the compound inhibits HDACs within 3 h of treatment.

**Fig. 5:**
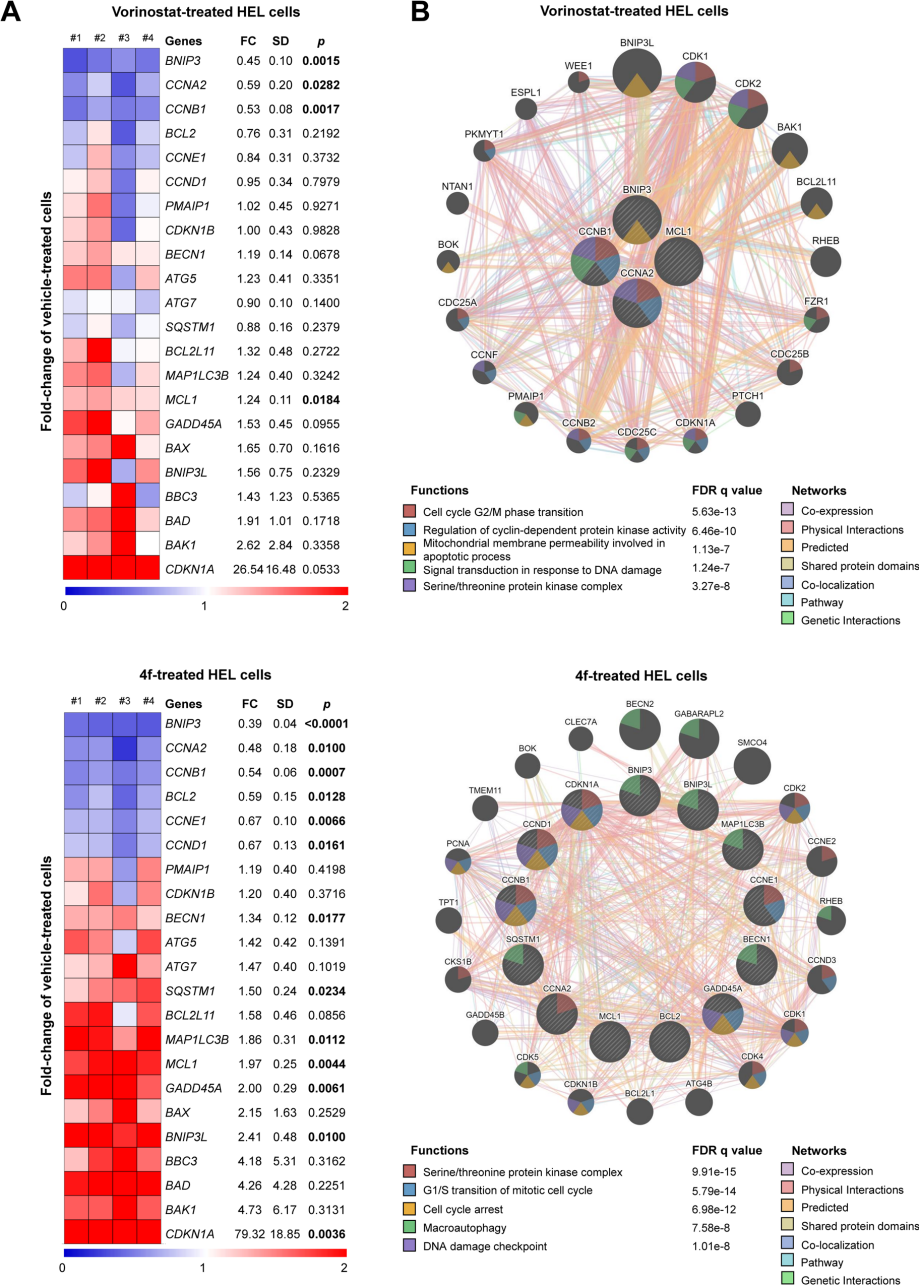
HDAC inhibitors promote a molecular network associated with cell cycle arrest, autophagy, and DNA damage response in HEL cells. **A** Quantitative PCR assay for a panel of key genes involved in proliferation, cell cycle progression, apoptosis, DNA damage, and autophagy was performed. The data presented represents the average of at least four independent experiments. Heatmap showing the gene expression profile of HEL cells treated with vehicle, vorinostat (1 μM), or **4** **f** (1 μM) for 24 h. Blue indicates decreased mRNA levels, while red indicates increased mRNA levels, normalized to vehicle-treated cells (*n* = 4). Fold-change (FC) relative to vehicle-treated cells, standard deviation (SD), and *p* values are described using the Student’s *t* test. **B** A gene network for vorinostat- or 4f-modulated genes was constructed using the GeneMANIA database (https://genemania.org/). Genes significantly modulated are represented as crosshatched circles, while interacting genes added by the software are shown as non-crosshatched circles. The main biological interactions, associated functions, and false discovery rate (FDR) q-values are detailed in the Figure.

Since HEL cells were more sensitive to **4** **f**, we selected this model for further molecular analyses. We investigated a broad panel of genes involved in cell cycle, apoptosis, DNA damage, and autophagy. Both vorinostat and **4** **f** modulated the genes *BNIP3*, *CCNB1*, *CCNA2*, and *MCL1* (Fig. 5A). Notably, HEL cells treated with **4** **f** showed a greater number of modulated genes compared to those treated with vorinostat. The biological processes and signaling pathways associated with vorinostat include cycle G_2_/M phase transition, regulation of cyclin-dependent protein kinase activity, mitochondrial membrane permeability involved in apoptotic process, signal transduction in response to DNA damage, and serine/threonine protein kinase complex. In contrast, **4** **f** was associated with pathways including serine/threonine protein kinase complex, G_1_/S transition of mitotic cell cycle, cell cycle arrest, macroautophagy, and DNA damage checkpoint (all FDR < 0.05, Fig. 5B).

### HDAC inhibition by 4 f potentiates apoptosis-induced by venetoclax in AML cells

Given the potential of **4** **f** as an antileukemic agent, we sought to evaluate whether this compound could be a better drug for clinical use in acute leukemias. To further refine this strategy, we first evaluated HDAC expression in AML, and observed that *HDAC2*, *HDAC6*, and *HDAC8* are overexpressed in AML patients when compared to healthy donors (Supplementary Fig. 4). Using data from ex vivo assays of the AML cohort of the Beat AML study, we identified a set of drugs in which high expression of *HDAC2*, *HDAC6*, and *HDAC8* would be associated with therapy resistance, with venetoclax being recurrent for *HDAC2* and *HDAC6* (Supplementary Fig. 5). Given the relevance of venetoclax for AML therapy [20], we decided to further investigate the combination of HDAC inhibitors with venetoclax in AML models with drug resistance.

**Fig. 6:**
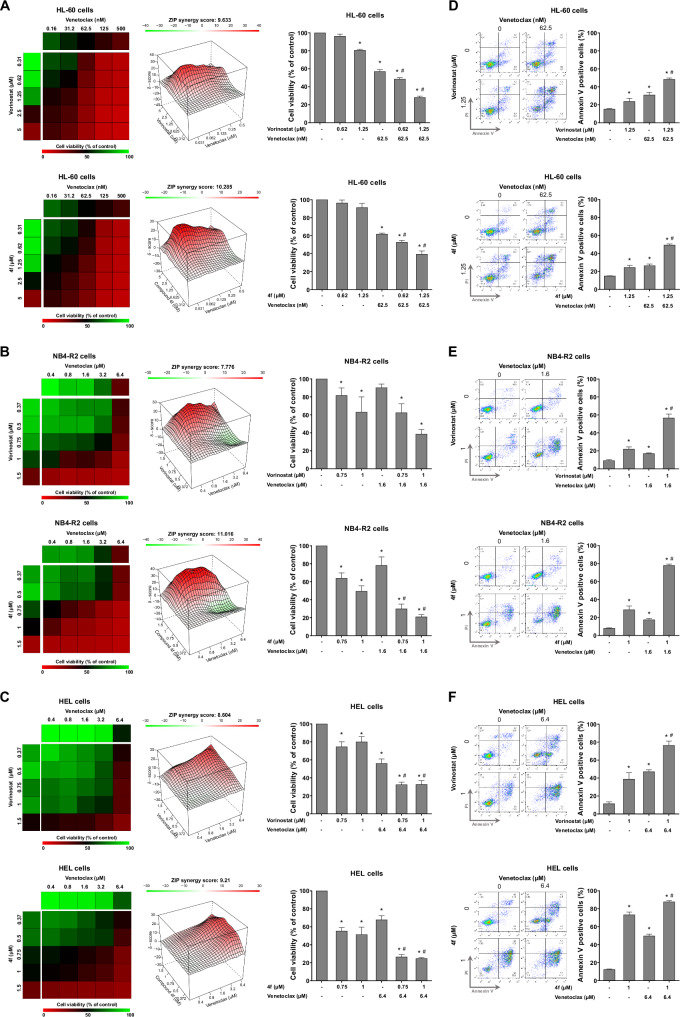
Compound 4 f acts synergistically on venetoclax-induced apoptosis and is more effective than vorinostat in acute myeloid leukemia (AML) cells. Dose-response cytotoxicity for vorinostat plus venetoclax and **4** **f** plus venetoclax was analyzed by methylthiazolethrazolium (MTT) assay in HL-60 (**A**), NB4-R2 (**B**) and HEL cells (**C**). AML were exposure to vehicle or graded concentrations of venetoclax and vorinostat or **4** **f** alone or in combination with each other for 48 h, as indicated. Values are expressed as the percentage of vehicle-treated cells. The ZIP synergy score was calculated using the SynergyFinder software (https://synergyfinder.fimm.fi/). Bar graphs highlight context-relevant combinations. *p < 0.05 treatment versus vehicle and ^#^*p* < 0.05 monotherapy versus combined therapy; ANOVA test and Bonferroni post-test. Results are shown as the mean of at least three independent experiments. HL-60 (**D**), NB4-R2 (**E**), and HEL (**F**) cells were labeled with APC-annexin V/propidium iodide (PI) being treated with vehicle or venetoclax and vorinostat or **4** **f** alone or in combination with each other for 48 h. Representative dot plots are shown for each condition. The upper and lower right quadrants (Q2 plus Q3) cumulatively contain the cell death population (annexin V+ cells). Bar graphs represent the mean ± SD of at least three independent experiments. The *p* values and cell lines are indicated in the graphs; *p < 0.05 treatment versus vehicle and ^#^*p* < 0.05 monotherapy versus combined therapy; ANOVA test and Bonferroni post-test.

In HL-60 (venetoclax-sensitive), HEL and NB4-R2 cells (intrinsically resistant to venetoclax) the combination of vorinostat or **4f** with venetoclax potentiated the reduction in viability (Fig. 6A–C) and the induction of apoptosis in all models tested (Fig. 6D–F).

## Discussion

In the USA, the incidence of AML is approximately 4.3 per 100,000 people per year, with a median diagnosis age of 65–72 years in Western countries, making it the most common type of acute leukemia among hematologic malignancies [21]. Treatment approaches are influenced by patient characteristics such as age, comorbidities, and performance status [22, 23]. It is crucial to determine the molecular profile of AML to select the most appropriate treatment. Standard induction chemotherapy involves cytarabine combined with anthracyclines or alternative agents to achieve complete remission, followed by consolidation therapy, often including high dose cytarabine or hematopoietic stem cell transplantation in eligible patients. Unfortunately, older patients are typically not eligible for intensive chemotherapy, and only 30% of these patients survive for five years (excluding the acute promyelocytic leukemia subtype). Outcomes for relapsed/refractory AML patients are poor, with less than 10% overall survival over three years [22–25]. These concerning clinical outcomes underscore the pressing need for new therapies or drug combinations to improve the treatment efficacy of acute leukemias.

Here in, we explored the potential of novel hybrid kinase/HDAC inhibitors in a broad panel of blood cancers, both as monotherapy and in combination with venetoclax, a leading targeted therapy for AML [19, 26]. HDACs are a group of enzymes classified into four classes, responsible for deacetylating histones, and some types may deacetylate non-histone substrates. HDACs remove acetyl groups from the lysine tails of histones, restoring their positive charge, which creates a stronger interaction between histones and DNA (which carries a negative charge), resulting in more condensed chromatin and reduced access to transcription factors. Consequently, transcription of suppressor tumor-related genes is downregulated [27]. Multiple studies have demonstrated that histone modifications play a role in the pathogenesis of both solid tumors and hematologic malignancies, often associated with advanced disease and poor prognosis [27–31]. For example, in AML, HDACs are aberrantly recruited by oncogenic fusion proteins like PML::RARα, PLZF::RARα, or AML1::ETO, leading to gene silencing, which contributes to differentiation arrest and leukemogenesis [27–30, 32].

In the present study, hybrid kinase/HDAC inhibitors selectively reduced leukemia cell viability in several blood cancer models. The use of epigenetic inhibitors targeting HDAC in clinical settings began around 2006 with vorinostat for cutaneous T-cell lymphoma released [33]. Following vorinostat’s promising results, other HDAC inhibitors demonstrated preclinical efficacy, leading to rapid clinical development, with several entering Phase I-III trials and being approved for hematologic malignancies [34]. For instance, belinostat showed an overall response rate of 25% and was well-tolerated in patients with cutaneous T-cell lymphoma or peripheral T-cell lymphoma [35]. Panobinostat, another HDAC inhibitor, induced histone hyperacetylation within 4 h of administration in patients (Phase I study) and reduced peripheral blood blasts in 11 patients (Phase II study), confirming its efficacy as a single agent or in combination with other drugs [34, 36–38]. While these drugs have been successful in treating hematologic disorders, none of them function as hybrid kinase/HDAC inhibitors, which could offer more potent effects by targeting additional cancer-specific pathways.

In a previous study, hybrid inhibitors combining purine-benzohydroxamate with substituted anilines or heteroaryl amines were used to inhibit kinases (including JAK1, JAK2, JAK3, BTK, CDK4, SKY, TRKα, PI3Kα, and PI3Kδ) and HDACs (HDAC1 and HDAC6) in blood cancer cells. These novel molecules, synthesized by our research group, were tested and found to be potent and specific against leukemia and lymphoma, providing chemical diversity that enhances anticancer effects by interacting with both HDACs and kinases [19]. Cancer’s capacity for mutational plasticity, especially when a single target is inhibited, often leads to treatment resistance via mutations in the target or activation of compensatory mechanisms. This results in most tumors acquiring resistance within a few months [39–41]. Combination therapy involving two or more drugs that interact with multiple targets has shown success in overcoming resistance. Ongoing clinical studies, approved by the FDA, aim to evaluate the efficacy, toxicity, and safety of new multi-target drugs, including tyrosine kinase inhibitors. However, no HDAC inhibitor-tyrosine kinase inhibitor combinations have yet been approved, though they represent an important avenue for cancer treatment [42–45].

In the present study, the hybrid kinase/HDAC inhibitor **4** **f** showed a synergistic effect in venetoclax-resistant AML cell lines. Venetoclax, a selective BCL2 inhibitor, has shown promising responses in clinical studies and was approved by the FDA in 2020 for AML. Despite promising results, resistance often develops after months to years of treatment. BCL2 has been shown to mediate chemoresistance and enhance the survival of leukemic blast [46, 47], making new drug combinations essential [48–53]. For example, in a study of a large cohort of AML patients treated with venetoclax and azacitidine, higher rates of complete remission were observed compared to the control group [53]. Another study in newly diagnosed and relapsed/refractory FLT3-mutated AML patients showed high remission rates when venetoclax was combined with azacitidine and gilteritinib [54] (NCT04140487). DiNardo et al. reported high response rates in patients treated with venetoclax and FLAG-IDA (fludarabine, cytarabine, G-CSF, and idarubicin) in both newly diagnosed and relapsed/refractory AML cases [55]. Even with these positive results, older patients often experience higher toxicity levels, necessitating alternative therapies [22–24]. The combination of HDAC inhibitors like panobinostat with venetoclax has been shown to sensitize cytarabine-resistant AML cells and enhance the cytotoxicity of standard therapies in blood cancer experimental models [56–58]. The combination of belinostat and venetoclax in primary cells reduced cell viability, induced apoptosis, and increased mitochondrial priming in T-cell prolymphocytic leukemia [59]. Similarly, chidamide in combination with venetoclax decreased cell viability and induced apoptosis in AML cell lines and primary cells, showing tumor growth reduction and prolonged survival in mice [60]. These studies corroborate our findings and highlight the potential of combining HDAC inhibitors with venetoclax for more effective treatment [61].

Clinically, venetoclax-based therapies achieve favorable responses, such as complete remission, in approximately one-third of AML patients, highlighting the urgent need to address intrinsic resistance mechanisms [20]. HEL and NB4 cells were selected for this study due to their poor responsiveness to venetoclax, with NB4 cells exhibiting intermediate resistance and HEL cells demonstrating high resistance to this agent. Our findings suggest that even in cell models intrinsically resistant to BCL2 inhibitors, the addition of HDAC inhibitors can enhance the efficacy of these agents. Mechanistically, although HDAC inhibitors do not directly alter BCL2 expression, chromatin remodeling may regulate the expression of other BCL2 family members or tumor suppressor genes, collectively contributing to the observed effects. For example, in HEL cells, a decrease in *BCL2* expression was accompanied by increases in *BECN1* and *BNIP3L*. BECN1 sequesters BCL2, thereby reducing its anti-apoptotic function, while BNIP3L, a BH3-only pro-apoptotic protein, may act as a sensitizer to apoptosis. Similarly, vorinostat treatment elevated the expression of *BNIP3*, another BH3-only pro-apoptotic protein [62–64]. In addition, treatment with compound **4** **f** induced the expression of tumor suppressor genes involved in DNA damage responses, such as *GADD45A* and *CDKN1A*, further contributing to reduced cell viability [65, 66]. These findings underscore the potential of HDAC inhibitors to overcome intrinsic resistance to venetoclax by modulating pro-apoptotic pathways and tumor suppressor gene networks.

In a previous study, the kinases JAK2 and JAK3 emerged as potential targets of the new hybrid kinase/HDAC inhibitor being investigated in the current research [19]. Notably, higher potency was observed in HEL cells, which harbor a constitutively active JAK2^V617F^ mutation that drives activation of the JAK2/STAT, MAPK, and PI3K/AKT pathways, promoting cellular proliferation and survival [67, 68]. Our data indicates that HDACi induces a transient inhibition of STAT3/5 phosphorylation in HEL cells, which may explain its greater potency compared to other cell lines (Supplementary Fig. 6).

In summary, purine-benzohydroxamate compounds, particularly **4** **f**, exhibit potent antineoplastic effects across a range of hematological malignancies, selectively reducing cell viability in neoplastic cells while sparing normal leukocytes. These effects are mediated through apoptosis induction, cell cycle arrest, and inhibition of HDAC activity, with **4** **f** demonstrating superior efficacy compared to vorinostat in both colony formation and differentiation assays. Additionally, **4** **f** modulates key genes involved in apoptosis, DNA damage, and autophagy, leading to enhanced therapeutic responses. Notably, the combination of **4** **f** with venetoclax significantly potentiates the reduction in cell viability, overcoming venetoclax resistance in AML models, highlighting the potential of **4** **f** as a promising therapeutic strategy for resistant hematological malignancies.

## Material and methods

### Cell culture and inhibitors

A panel containing cellular models of hematological malignancies, including acute myeloid leukemia (AML), acute lymphoblastic leukemia (ALL), lymphoma, and multiple myeloma was used. U-937, HEL, K-562, KU812, Jurkat, Namalwa, Daudi, Raji, REH, U266, MM1.S, and MM1.R cells were provided by Prof. Sara Teresinha Olalla Saad (Hemocentro, University of Campinas, Brazil). OCI-AML3, Kasumi-1, HL-60, THP-1, MOLM-13, MV4-11, NB4, and NB4-R2 cells were provided by Prof. Eduardo Magalhães Rego (Faculdade de Medicina, University of São Paulo, Brazil). SET-2 cells were provided by Prof. Dr. Fabíola Attié de Castro (School of Pharmaceutical Sciences of Ribeirão Preto, University of São Paulo, Ribeirão Preto, Brazil). CEM and NALM6 cells were provided by Dr. Gilberto Carlos Franchi Junior (Universidade de Campinas, Campinas, Brazil). The cell lines were cultivated in culture medium recommended by the American Type Culture Collection (ATCC) or Deutsche Sammlung von Mikroorganismen und Zellkulturen (DSMZ), supplemented with fetal bovine serum and penicillin/streptomycin. The cells were maintained at 37°C, 5% CO_2_. Peripheral blood mononuclear cells from healthy donors (normal leukocytes) were obtained by Ficoll gradient (Sigma-Aldrich) according to the manufacturer’s instructions and cultured in RPMI-1640 medium containing 30% FBS, penicillin/streptomycin, and recombinant cytokines (Peprotech Inc., Rocky Hill, NJ, USA) (30 ng/mL IL-3, 100 ng/mL IL-7, 100 ng/mL FLT3 ligand, and 30 ng/mL SCF). The study was approved by the ICB-USP Human Research Ethics Committee (Protocol: 4423074; CAAE: 39510920.1.0000.5467). Vorinostat was obtained from Sigma-Aldrich (St. Louis, MO, USA). Compounds **4** **d,** **4e**, and **4** **f** were synthesized as previously described [19]. The compounds were diluted in dimethyl sulfoxide (DMSO) (Synth, Diadema, SP, Brazil) at 50 mM and stored at –20 °C. In the ‘vehicle condition (0 µM), cells were treated with an equivalent amount of DMSO. The chemical structures of HDACi are illustrated in Supplementary Fig. 7.

### Cell viability assay

Cell viability was measured using the MTT assay, according to the manufacturer’s instructions (Sigma-Aldrich, St. Louis, MI, USA). Briefly, 2 × 10^4^ (cell lines) or 2 × 10^5^ cells (primary cells) per well were cultured in a 96-well plate with the appropriate culture medium and increasing concentrations of the analyzed compounds. After 24, 48, and/or 72 h of incubation, 10 μL of a solution containing MTT at 5 mg/mL was added and incubated for 4 h at 37 °C. The reaction was then stopped by adding 100 μL of 0.1 N HCl in isopropanol. Cell viability was measured at an absorbance of 570 nm using an automatic plate reader. The experiments were performed with at least three replicates per condition, and at least three independent experiments were conducted for each condition. IC_50_ values were calculated using nonlinear regression analysis in GraphPad Prism 8 software (GraphPad Software, Inc., San Diego, CA, USA). For combined treatment analysis, HL-60 cells were exposed to graded doses of vorinostat (0.31, 0.62, 1.25, 2.5, and 5 μM) and venetoclax (0.16, 31.3, 62.5, 125, and 500 nM) or **4** **f** (0.31, 0.62, 1.25, 2.5, and 5 μM) and venetoclax (0.16, 31.3, 62.5, 125, and 500 nM) alone or in combination for 48 h. NB4-R2 or HEL cells were exposed to graded doses of vorinostat (0.25, 0.5, 0.75, 1, and 1.5 μM) and venetoclax (0.4, 0.8, 1.6, 3.2, and 6.4 µM) or **4** **f** (0.25, 0.5, 0.75, 1, and 1.5 μM) and venetoclax (0.4, 0.8, 1.6, 3.2, and 6.4 µM) alone or in combination for 48 h. Data were illustrated using the Multiple Experiment Viewer (MeV) 4.9.0 software (http://www.tm4.org/mev/). The ZIP synergy score was calculated using the SynergyFinder software (https://synergyfinder.fimm.fi/).

### Cell death assay

HEL, MOLM-13, NB4-R2, or THP-1 cells (1 × 10^5^ per well) were seeded in 24-well plates with vehicle, vorinostat, **4** **d,** **4e**, and **4** **f** (0.75, 1, 1.5, or 2 μM) for 24 h.

For combined treatment, cells were exposed to vorinostat or **4** **f** (HL-60: 1.25 μM, NB4-R2 and HEL: 1 μM) and venetoclax (HL-60: 62.5 nM, NB4-R2: 1.6 μM, and HEL: 6.4 μM) alone or in combination for 48 h. The cells were then washed with ice-cold phosphate-buffered saline (PBS) and resuspended in a binding buffer containing 1 μg/mL propidium iodide (PI) and 1 μg/mL APC-labeled annexin V. All specimens were analyzed by flow cytometry (FACSCalibur; Becton Dickinson, Franklin Lakes, NJ, USA) after incubation for 15 min at room temperature in a light-protected area. Ten thousand events were recorded for each sample.

### Cell cycle assay

HEL, MOLM-13, NB4-R2, or THP-1 cells (1 × 10^5^ per well) were seeded in 12-well plates with vehicle, vorinostat, or **4** **f** (0.75, 1, or 1.5 μM), and then harvested after 24 h. The cells were fixed with 70% ethanol and stored at 4 °C for at least 4 h. The fixed cells were then stained with PBS containing 20 μg/mL propidium iodide (PI) and 10 μg/mL RNase A for 30 min at room temperature in a light-protected area. DNA content distribution was determined using flow cytometry (FACSCalibur; Becton Dickinson) and analyzed with FlowJo software (TreeStar, Inc.).

### Autonomous colony formation assay

Colony formation assays were performed in semi-solid methylcellulose medium (1 × 10^3^ cells/mL; MethoCult 4230; StemCell Technologies Inc., Vancouver, BC, Canada) in the presence of vehicle, vorinostat, or **4** **f** (0.25, 0.5, 1, 1.5, and 2 μM). After ten days of culture, colonies were detected by adding MTT solution (5 mg/mL). Images were acquired using the G:BOX Chemi XX6 gel documentation system (Syngene, Cambridge, United Kingdom) and analyzed with ImageJ software (US National Institutes of Health, Bethesda, MD, USA).

### Cell differentiation analysis

NB4-R2 and THP-1 cells were treated with the vehicle, vorinostat or **4** **f** for 96 h and subjected flow cytromtery and panoptic staining. After treatment, cells were collected and resuspended in PBS containing 5 μL of anti-CD11b-PE. Following incubation in the dark for 30 min at room temperature, the samples were washed with PBS and analyzed on a FACSCalibur. Ten thousand events were acquired for each sample. For cytospin, a total of 1 × 10^5^ cells were adhered to microscopic slides using a cytospin (Serocyte, model 2400, FANEM, Guarulhos, Brazil) for subsequent Rosenfeld staining. Morphological analysis of the nucleus and cytoplasm was visualized using a Leica DM 2500 optical microscope, and images were acquired with LAS V4.6 software (LEICA, Bensheim, Germany).

### Western blot analysis

Total protein extraction was performed using a buffer containing 100 mM Tris (pH 7.6), 1% Triton X-100, 2 mM PMSF, 10 mM Na_3_VO_4_, 100 mM NaF, 10 mM Na_4_P_2_O_7_, and 4 mM EDTA. Equal amounts of protein (15 μg) from the samples were subjected to SDS-PAGE in an electrophoresis device, followed by electrotransfer of the proteins to nitrocellulose membranes. The membranes were blocked with 5% non-fat dry milk and incubated with specific primary antibodies diluted in blocking buffer, followed by secondary antibodies conjugated to horseradish peroxidase (HRP). Western blot analysis was performed using a SuperSignal™ West Dura Extended Duration substrate system (Thermo Fisher Scientific, Waltham, MA, USA) and a G:BOX Chemi XX6 gel documentation system (Syngene). Antibodies against acetyl-histone H3 Lys9 (#9649), histone H3 (#4499), acetyl-α-tubulin Lys40 (#5335), α-tubulin (#2144), and GAPDH (#5174) were obtained from Cell Signaling Technology (Danvers, MA, USA). Cropped gels retain important bands, but entire gel images are available in Supplementary Fig. 8.

### Quantitative RT-PCR (qRT-PCR)

Total RNA was extracted using TRIzol reagent (Thermo Fisher Scientific), and cDNA was synthesized from 1 μg RNA using a High-Capacity cDNA Reverse Transcription Kit (Thermo Fisher Scientific). Quantitative PCR (qPCR) was performed using a QuantStudio 3 Real-Time PCR System in conjunction with a SYBR Green system (Thermo Fisher Scientific). *HPRT1* and *ACTB* were used as reference genes. Relative quantification values were calculated using the 2^-ΔΔCT^ equation [69]. Expression of cell cycle-, apoptosis-, DNA damage-, and autophagy-related genes (Supplementary Table 1) was investigated in HEL cells following exposure to vehicle, vorinostat (1 μM), or **4** **f** (1 μM) for 24 h. A heatmap was generated using the Multiple Experiment Viewer (MeV) 4.9.0 software (http://mev.tm4.org). Differentially expressed genes with *p* > 0.05 were used for network construction using the GeneMANIA database (https://genemania.org/).

### Bioinformatics

*HDAC1*, *HDAC2*, *HDAC3*, *HDAC4*, *HDAC5*, *HDAC6*, *HDAC7*, *HDAC8*, *HDAC9*, *HDAC10*, and *HDAC11* mRNA expression data from healthy donors (*n* = 13) and AML (*n* = 577) were obtained from Amazonia! database 2008 [70], which were generated using cDNA microarrays Affytmetrix HGU133 plus 2.0 arrays. Data sets were cross-referenced using tumor-specific identification numbers. The numbers of individuals for each group are indicated. Correlation analysis was performed using the Spearman test and RStudio software (version 1.4.1717, RStudio, PBC) and the Corrplot plugin. The area under the curve (AUC) values from 165 drugs tested in ex vivo assays by the Beat AML study (n = 520) were utilized to explore the correlation between drug response and *HDAC2*, *HDAC6*, and *HDAC8* expression [71, 72].

### Statistical analysis

Statistical analyses were performed using GraphPad Prism 8 (GraphPad Software Inc.). Mann–Witney test, analysis of variance (ANOVA) and Bonferroni post-test, Student *t* test, or Spearmen correlation test were used, as appropriate. A *p* < *0.05* were statistically considered.

## Supplementary information

Supplementary Table 1

Supplementary Legends

Supplementary Figure 1

Supplementary Figure 2

Supplementary Figure 3

Supplementary Figure 4

Supplementary Figure 5

Supplementary Figure 6

Supplementary Figure 7

Supplementary Figure 8

Full length uncropped original Western blots

## Data Availability

The data generated or analyzed in this study during the current study are available from the corresponding author on reasonable request.
