# Supplementary material for: Enhancing venetoclax efficacy in leukemia through association with HDAC inhibitors
Source: Cell Death Discov. 2025 Apr 6;11:147. doi: 10.1038/s41420-025-02446-4 (PMC11972356; doi:10.1038/s41420-025-02446-4)
Supplement: Supplementary file 2 — Supplementary Legends [file 41420_2025_2446_MOESM2_ESM.docx]

**Enhancing venetoclax efficacy in leukemia through association with HDAC inhibitors**

Jorge Antonio Elias Godoy Carlos^1^, Mauricio Temotheo Tavares^2,3,4^, Keli Lima^1,5^, Larissa Costa de Almeida^1^, Karoline de Barros Waitman^2^, Leticia Veras Costa-Lotufo^1^, Roberto Parise-Filho^2^, João Agostinho Machado-Neto^1^

^1^ Department of Pharmacology, Institute of Biomedical Sciences, University of São Paulo, São Paulo, Brazil

^2^ Department of Pharmacy, Faculty of Pharmaceutical Science, University of São Paulo, São Paulo, Brazil

^3^ Department of Cancer Biology, Dana-Farber Cancer Institute, Boston, MA, 02115, United States

^4^ Department of Biological Chemistry and Molecular Pharmacology, Harvard Medical School, Boston, MA, 02115, United States

^5^ Laboratory of Medical Investigation in Pathogenesis and Targeted Therapy in Onco-Immuno-Hematology (LIM-31), Department of Internal Medicine, Hematology Division, Faculty of Medicine, University of São Paulo, São Paulo, Brazil

**Supplementary Figure Legends**

**Supplementary Figure 1. Purine-benzohydroxamate compounds reduce cell viability in a time- and concentration-dependent manner in acute myeloid leukemia (AML) cells.** Dose- and time-response cytotoxicity was analyzed using the methylthiazol tetrazolium (MTT) assay in HEL, NB4-R2, MOLM-13, and THP-1 AML cell lines treated with vehicle or increasing concentrations of vorinostat, **4d**, **4e**, or **4f** (0.08, 0.4, 2, and 4 µM) for 24, 48, and 72 hours. The bar graph represents the mean ± SD of at least three independent experiments. IC_50_ values for each compound are provided. The p-values and cell lines are indicated in the figure; **p* < 0.05, ***p* < 0.01, ****p* < 0.0001; ANOVA and Bonferroni post-test.

**Supplementary Figure 2. Novel HDAC inhibitors induce apoptosis in acute myeloid leukemia cells.** Representative dot plots are shown for each condition. The upper and lower right quadrants (Q2 + Q3) collectively represent the apoptotic cell population (annexin V+ cells).

**Supplementary Figure 3. Compound 4f induces cell differentiation markers in acute myeloid leukemia (AML) cells.** NB4-R2 and THP-1 cells were treated with vehicle, vorinostat, or **4f** for 96 hours. **(A)** CD11b expression was analyzed by flow cytometry, and a histogram is presented. The bar graph represents the mean ± SD of at least three independent experiments; ****p* < 0.0001 (ANOVA and Bonferroni post-test). **(B)** Cytospin analysis revealed a change in the nuclei/cytoplasm ratio compared to vehicle-treated cells. Additionally, cytoplasmic vacuoles were observed after exposure to the drugs. Image magnification 1000×.

**Supplementary Figure 4. *HDAC2*, *HDAC6*, and *HDAC8* are highly expressed in acute myeloid leukemia (AML) patients.** The mRNA levels of *HDAC1*, *HDAC2*, *HDAC3*, *HDAC4*, *HDAC5*, *HDAC6*, *HDAC7*, *HDAC8*, *HDAC9*, *HDAC10*, and *HDAC11* were measured in samples from healthy donors (n = 13) and AML patients (n = 577) from the Amazonia! database (2008). The Y-axis represents gene expression data generated from cDNA microarray analysis using Affymetrix HGU133 Plus 2.0 arrays. Datasets were cross-referenced using tumor-specific identification numbers, and the number of subjects for each group is indicated. **p* < 0.05, ***p* < 0.01, ****p* < 0.0001; Mann-Whitney test. A correlation diagram between the expressions of all HDACs was constructed and is illustrated in the figure.

**Supplementary Figure 5. Impact of *HDAC2*, *HDAC6*, and *HDAC8* expression on drug sensitivity in *ex vivo* assays of acute myeloid leukemia (AML) cells.** Drug sensitivity was analyzed in relation to *HDAC2*, *HDAC6*, and *HDAC8* mRNA levels in *ex vivo* assays of AML samples from the Beat AML cohort. Drugs with *p* < 0.05 are indicated using the Spearman correlation test.

**Supplementary Figure 6. HDAC inhibitors target JAK2/STAT signaling in HEL cells.** Western blot analysis was performed to detect phospho(p)-STAT3 (Tyr705), STAT3, p-STAT5 (Tyr694), STAT5, α-tubulin or GAPDH in total cell extracts from **(A)** HEL, MOLM-13, NB4-R2, and THP-1 cells or **(B)** HEL cells treated with vehicle, vorinostat (1 µM), or **4f** (1 µM) for 0, 3, 6, 9, or 12 hours. Vorinostat was used as a reference drug. Cell lines are indicated in the Figure.

**Supplementary Figure 7. Chemical structure of the HDAC inhibitors used in the study.** The structures of vorinostat, **4d**, **4e**, and **4f** are illustrated.

**Supplementary Figure 8. Whole gels for Western blot analysis.** Figures associated with Western blot experiments and molecular weight are indicated.
