# Supplementary figures and images for: Enhancing venetoclax efficacy in leukemia through association with HDAC inhibitors
Source: Cell Death Discov. 2025 Apr 6;11:147. doi: 10.1038/s41420-025-02446-4 (PMC11972356; doi:10.1038/s41420-025-02446-4)

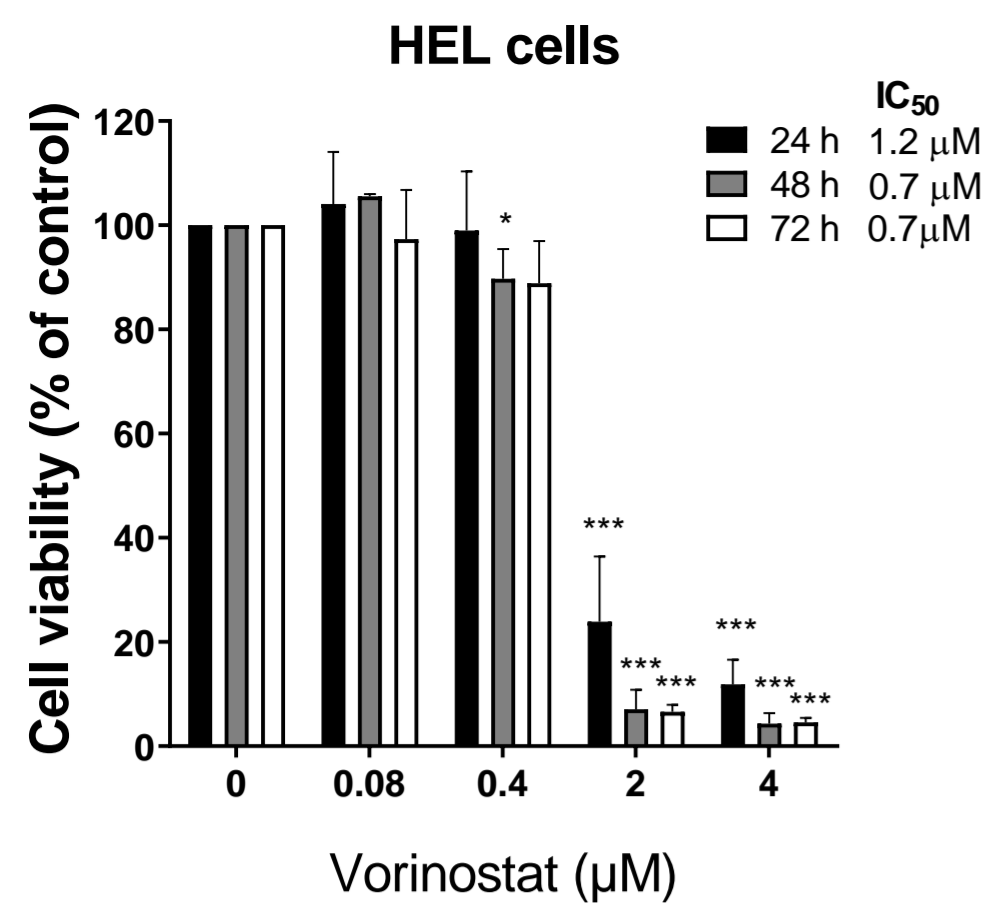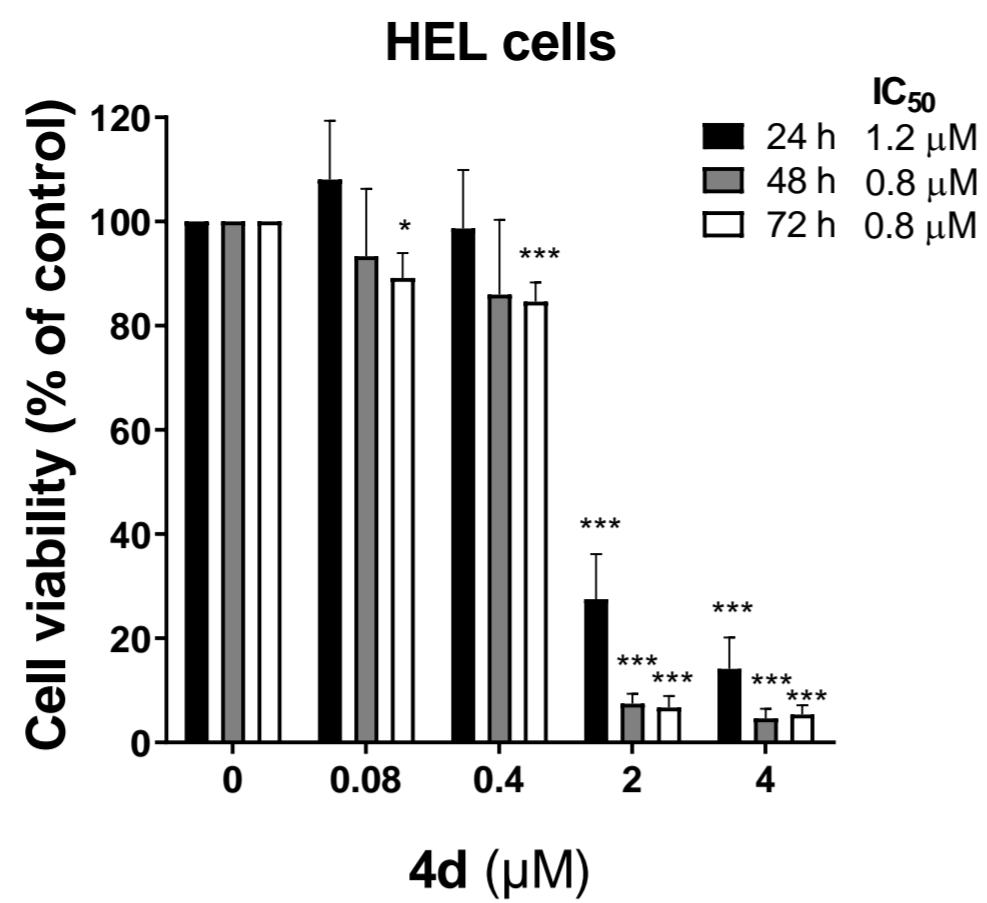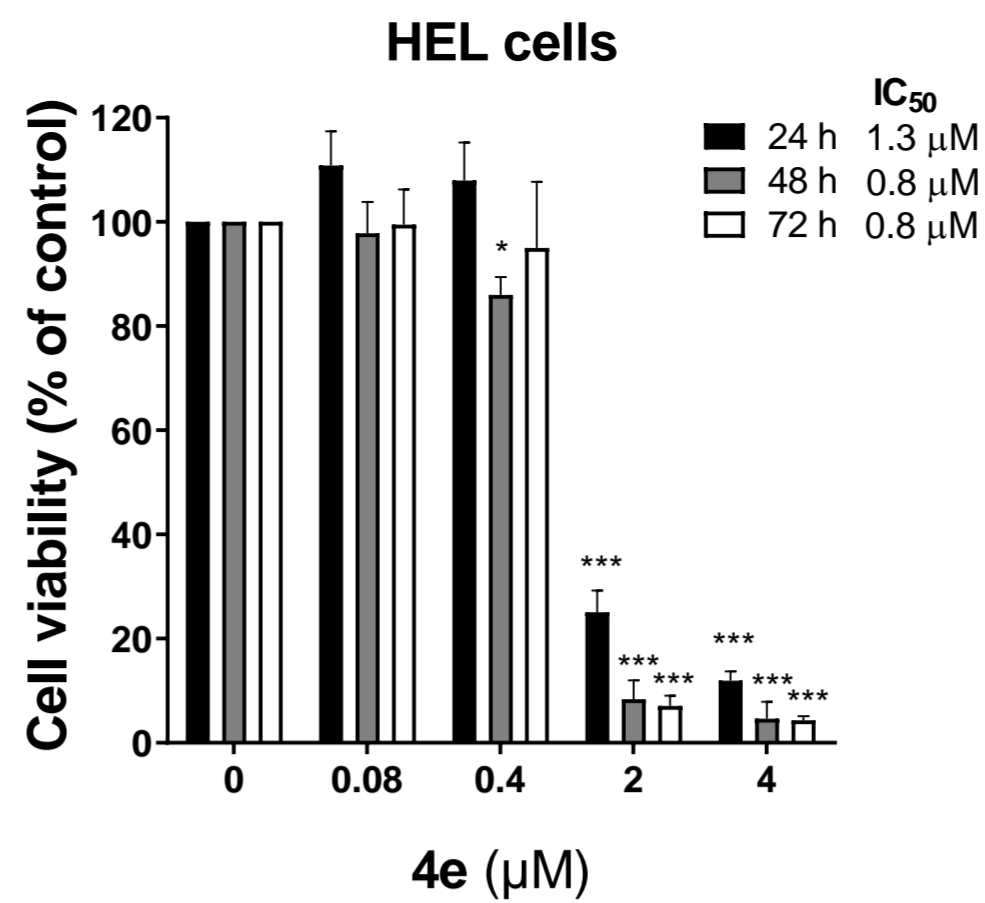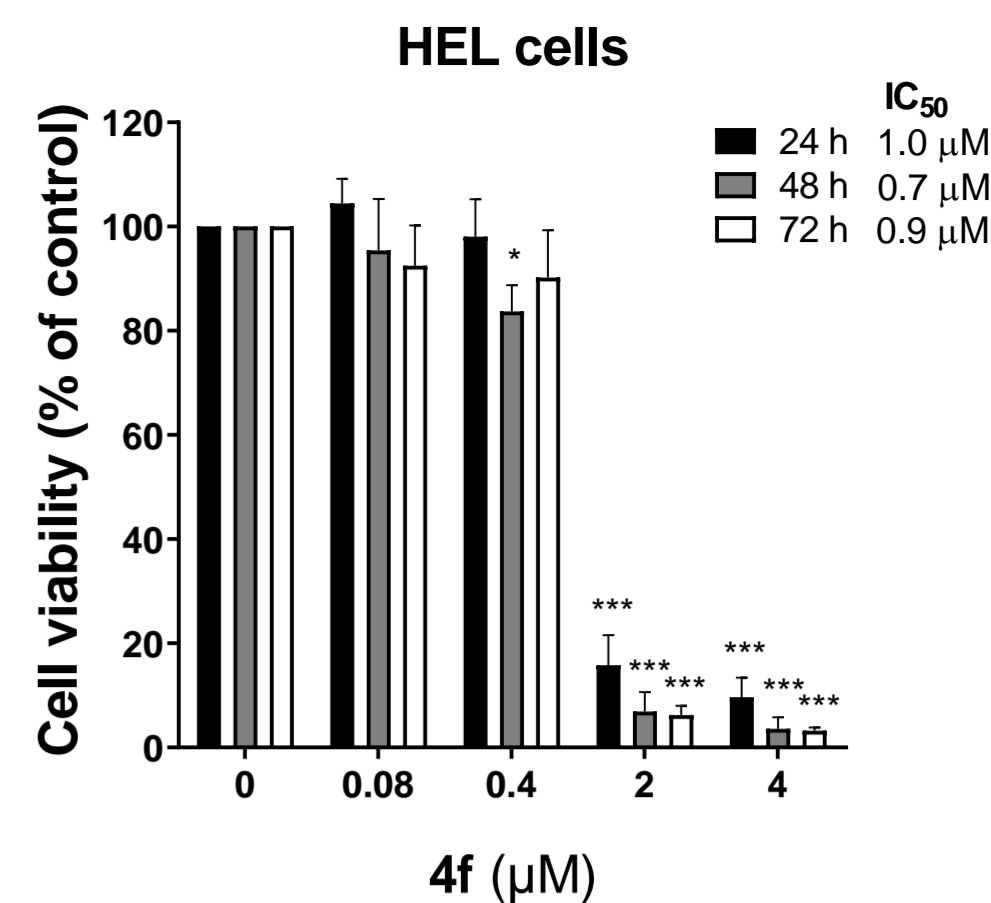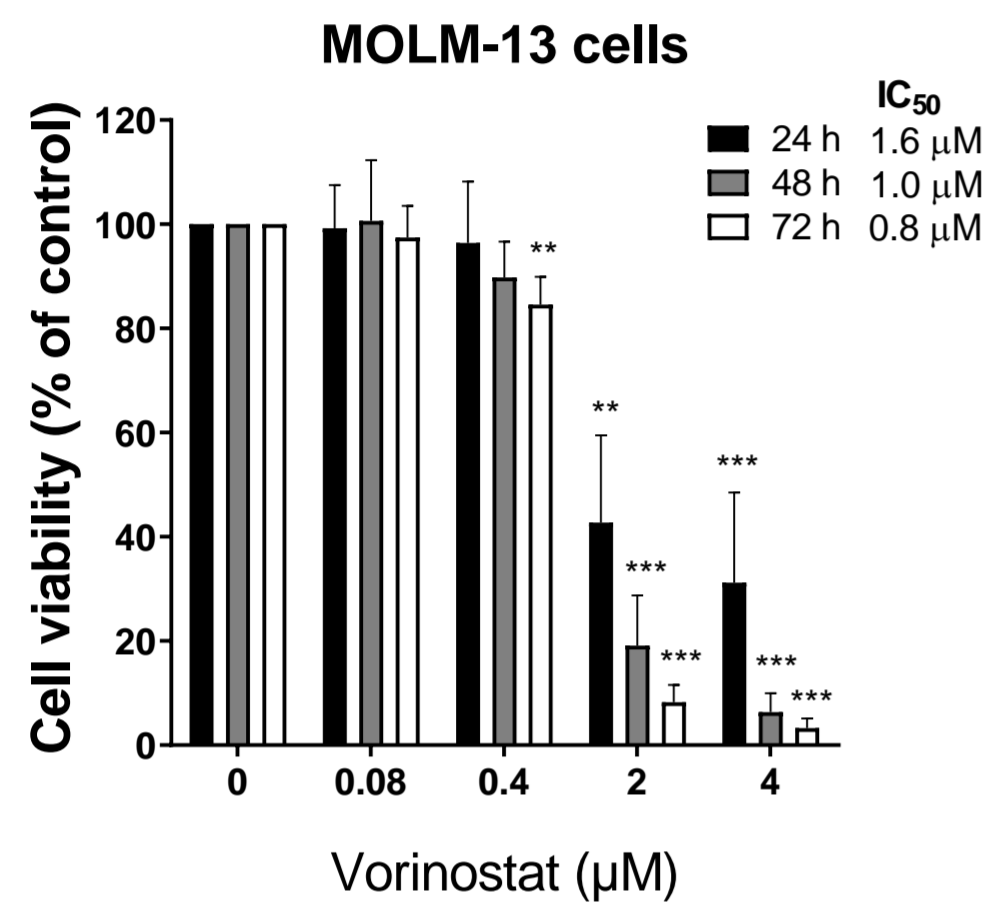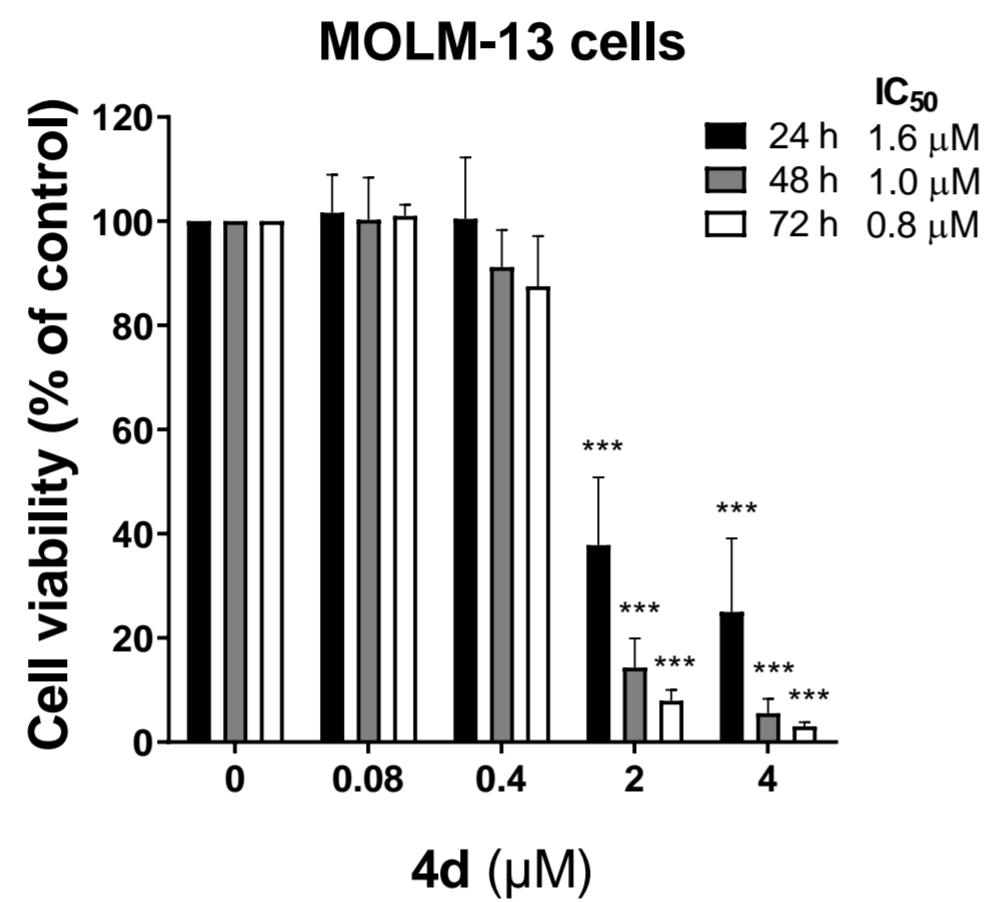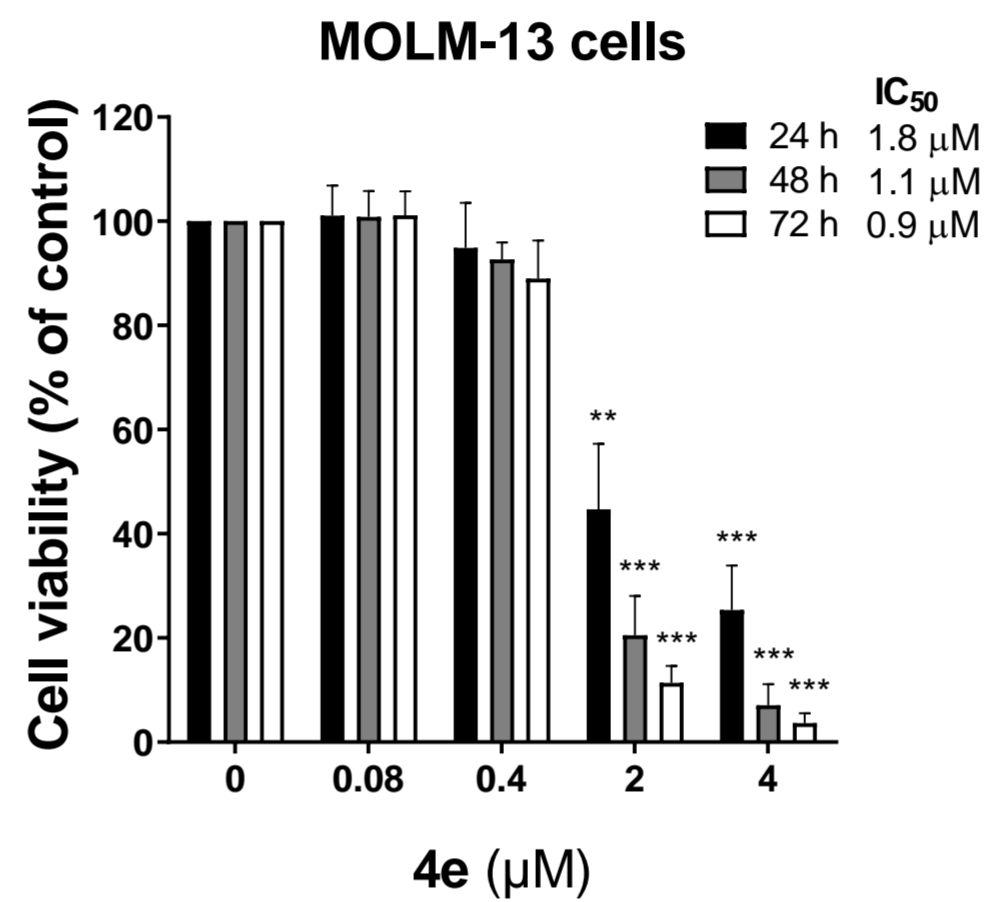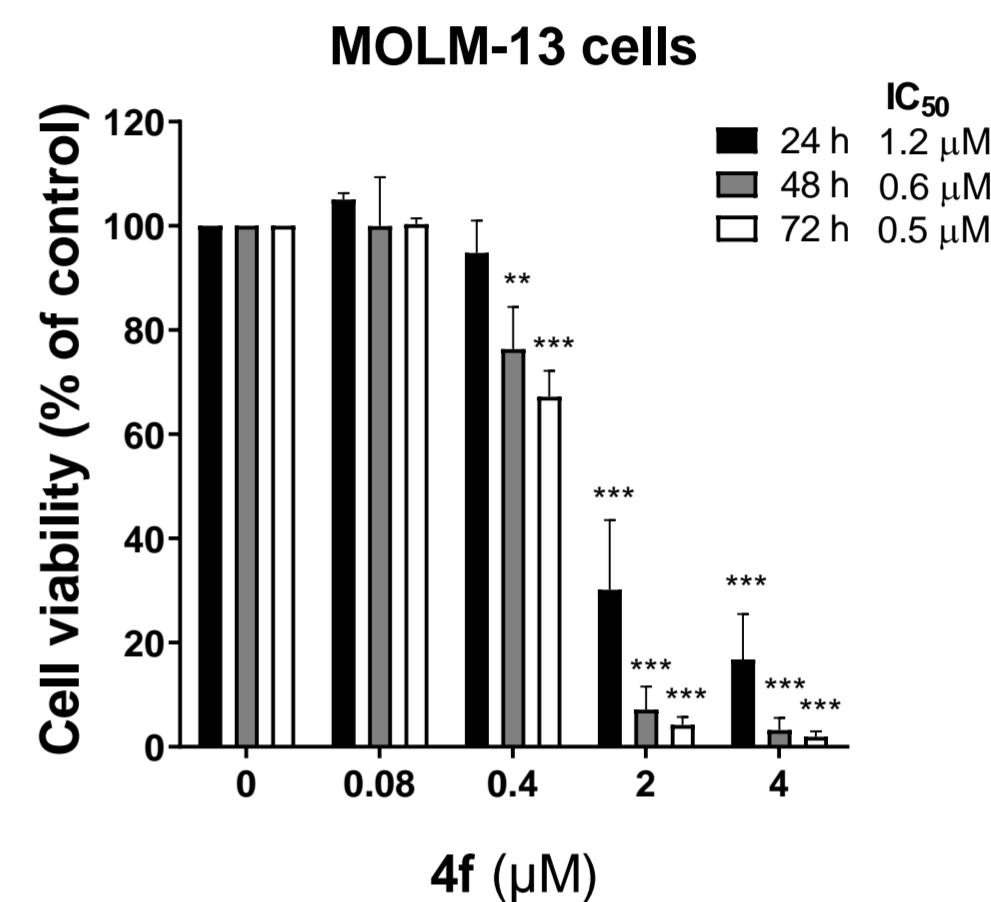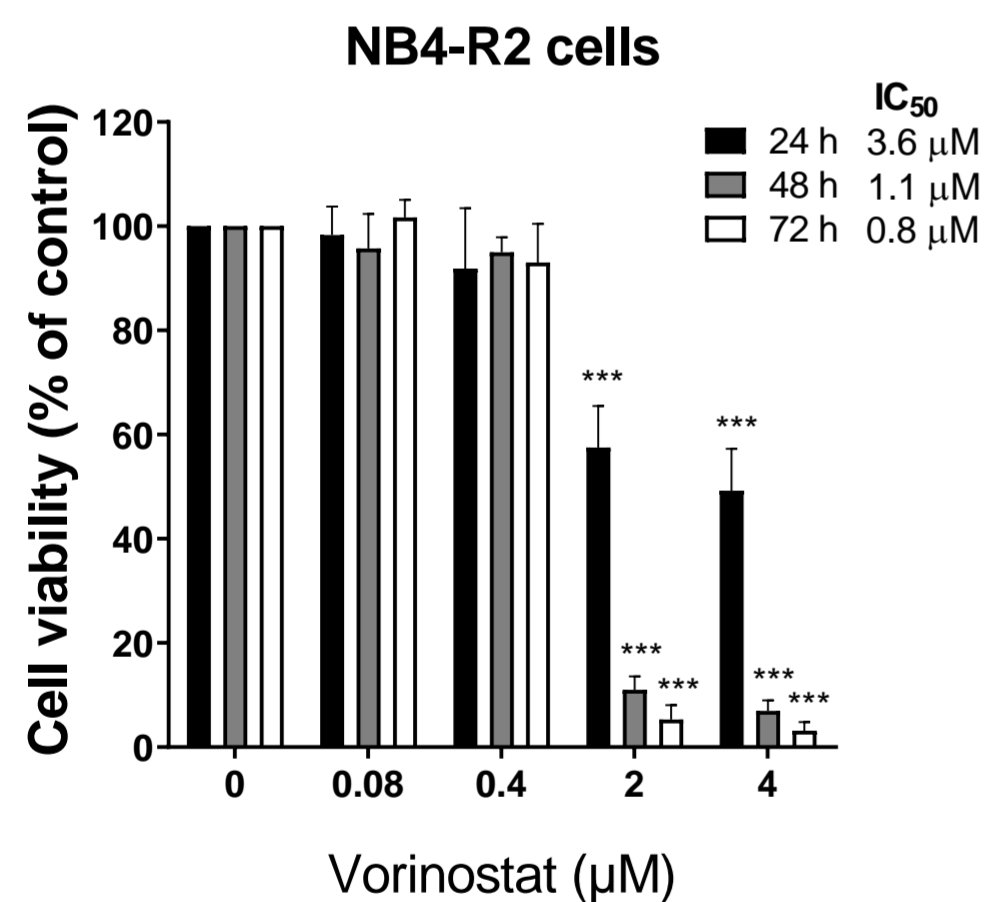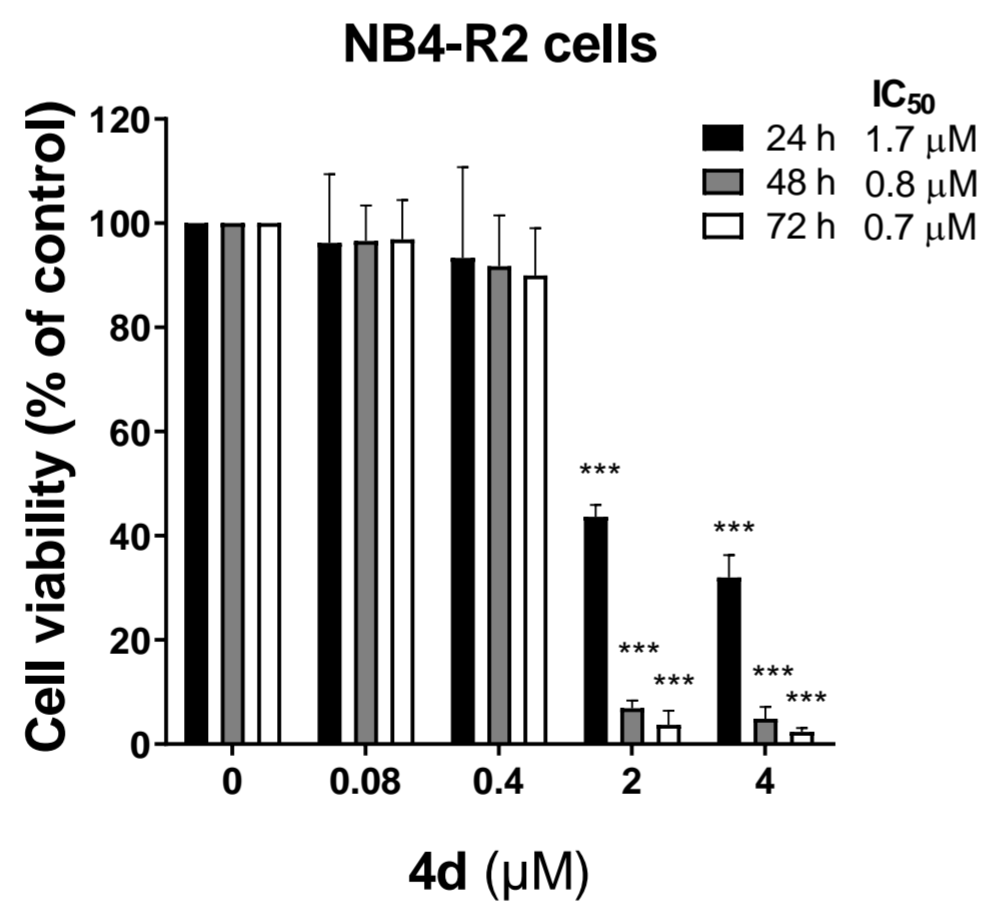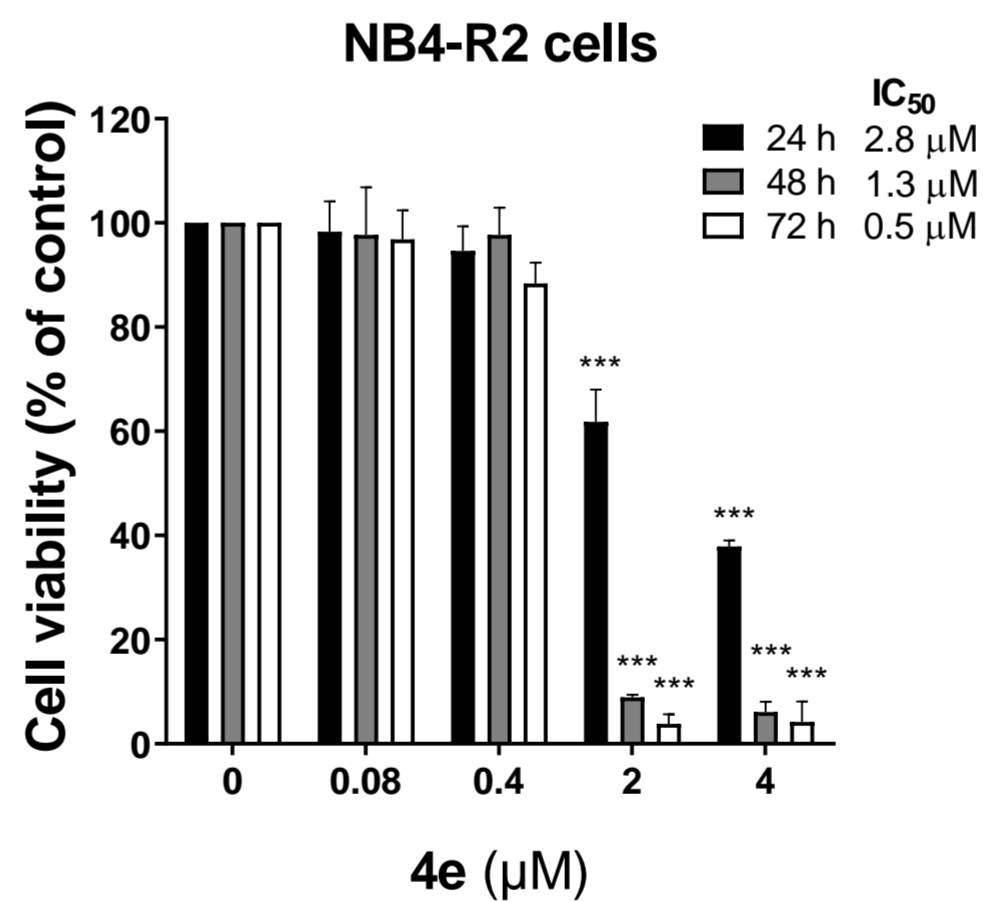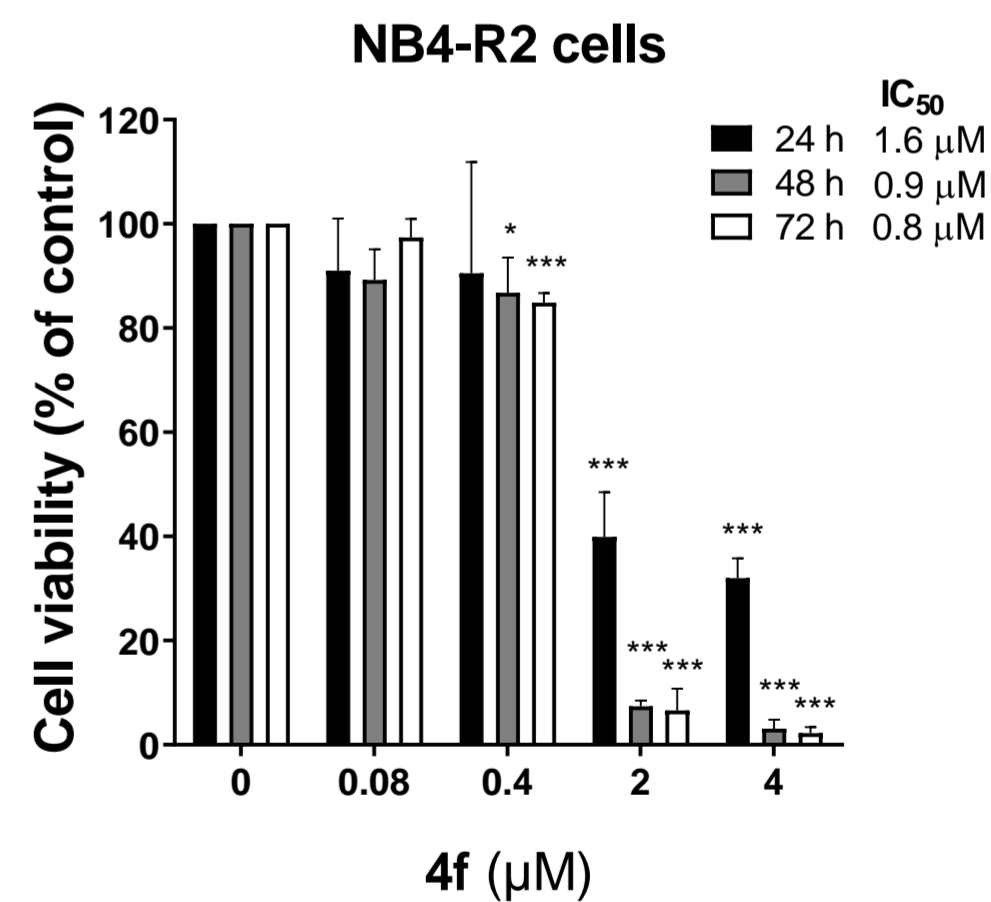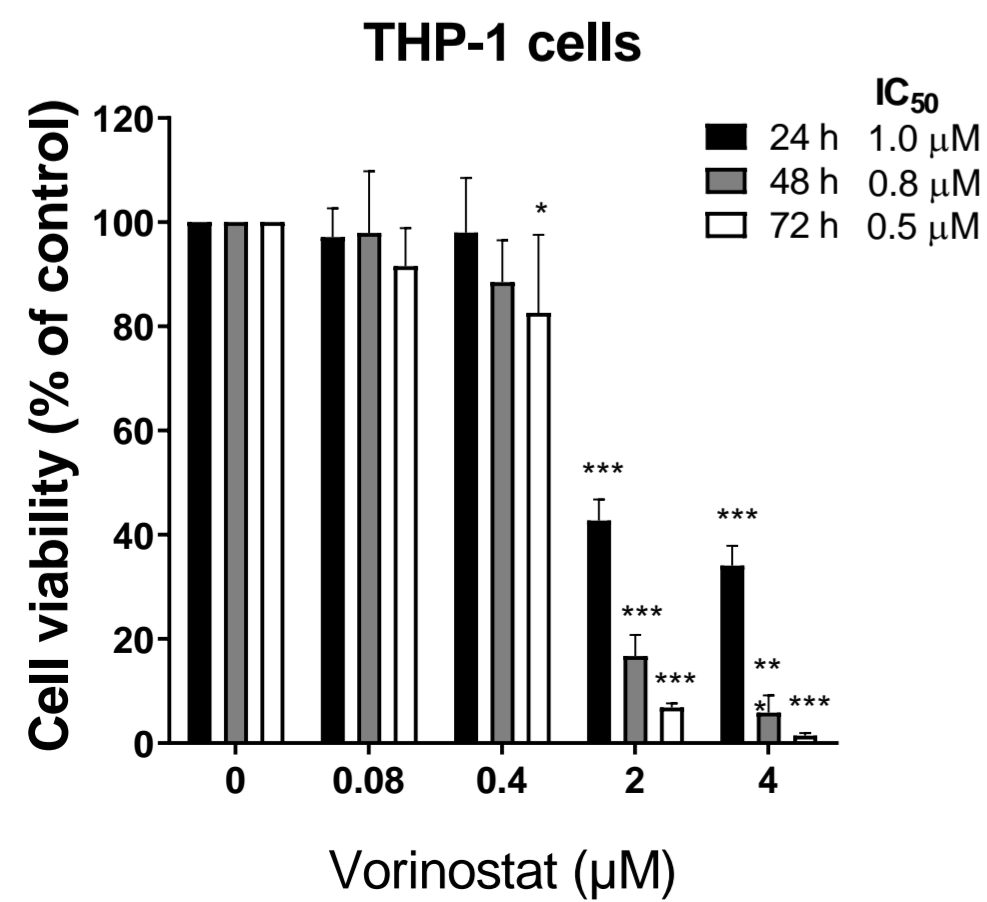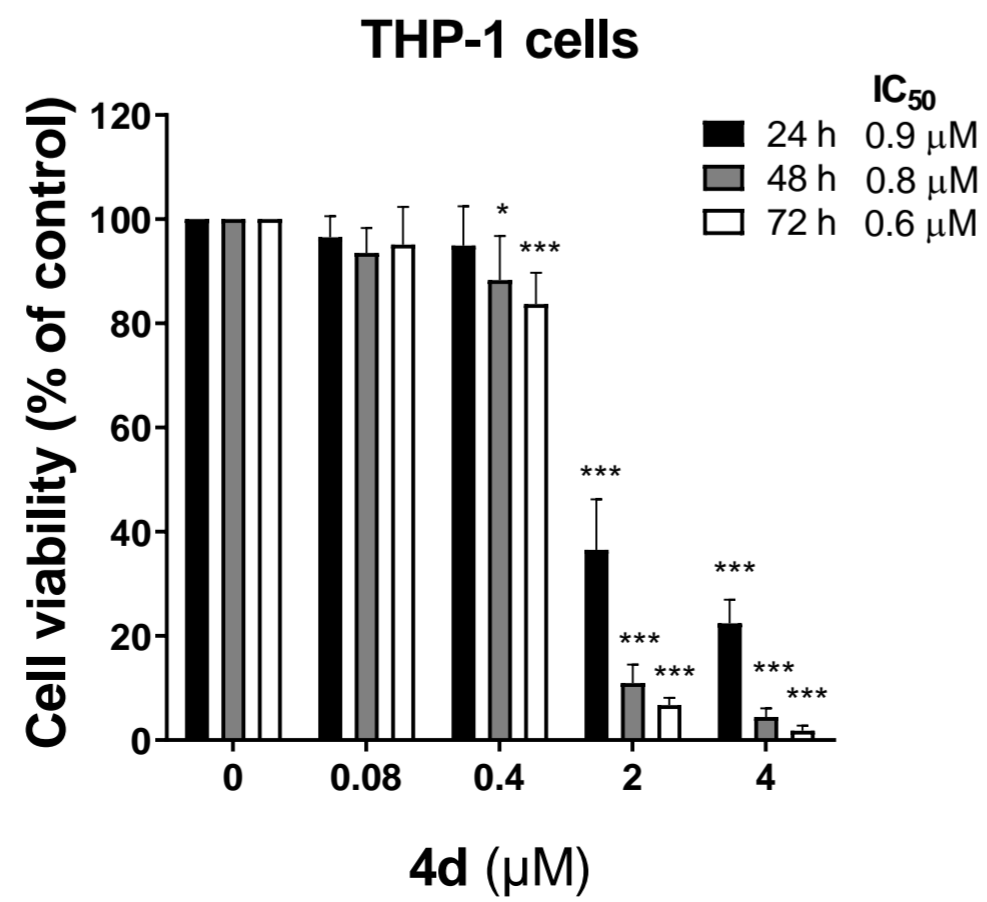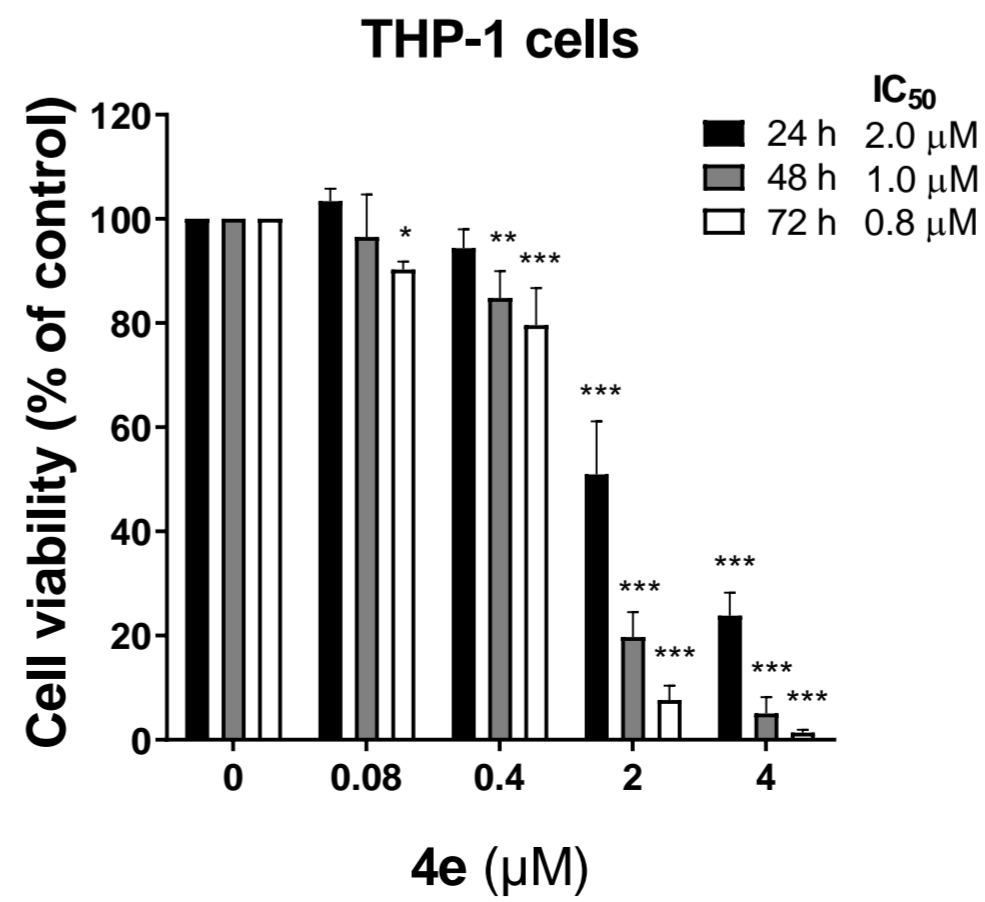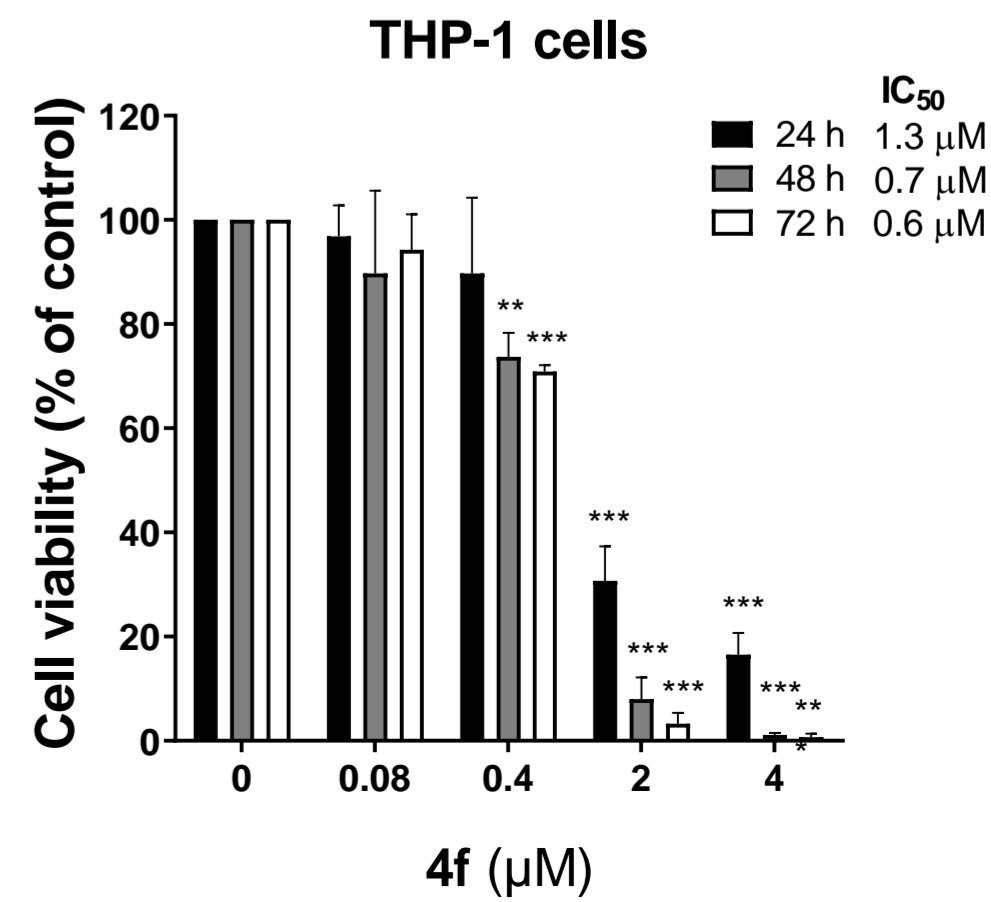

Supplement: Supplementary file 3 — Supplementary Figure 1 [file 41420_2025_2446_MOESM3_ESM.pdf]

**A****HEL cells**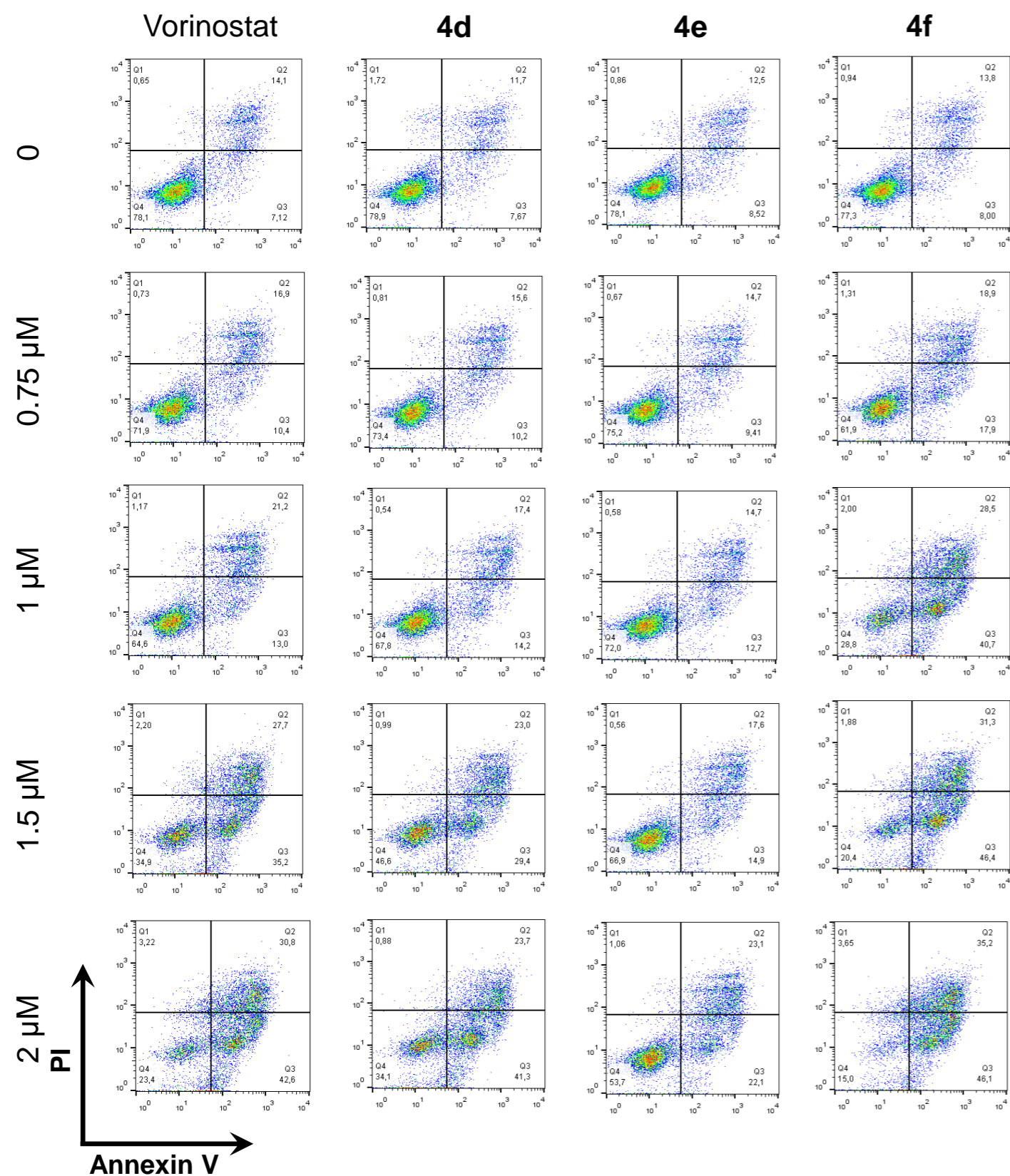**C****NB4-R2 cells**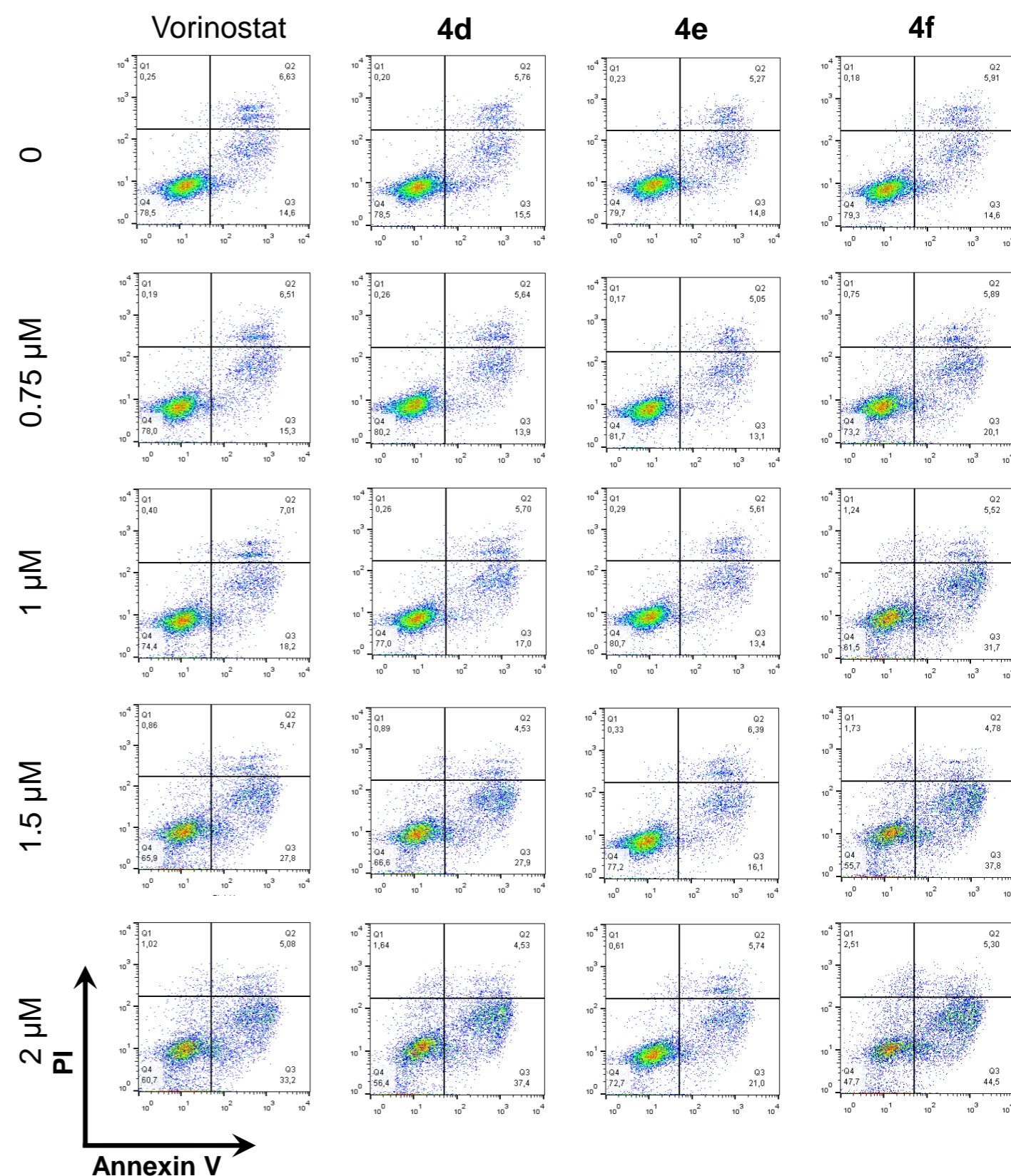**B****MOLM-13 cells**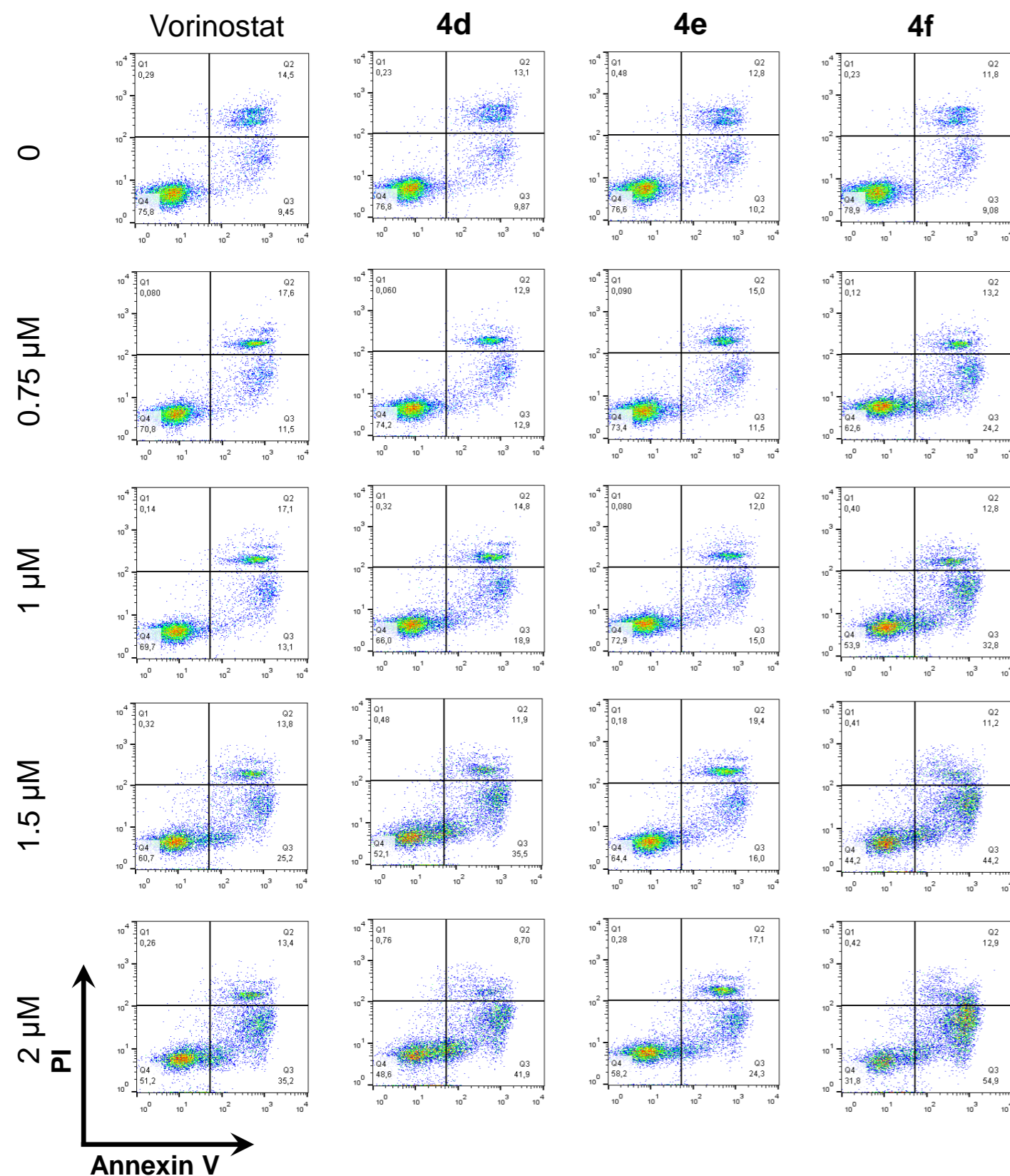**D****THP-1 cells**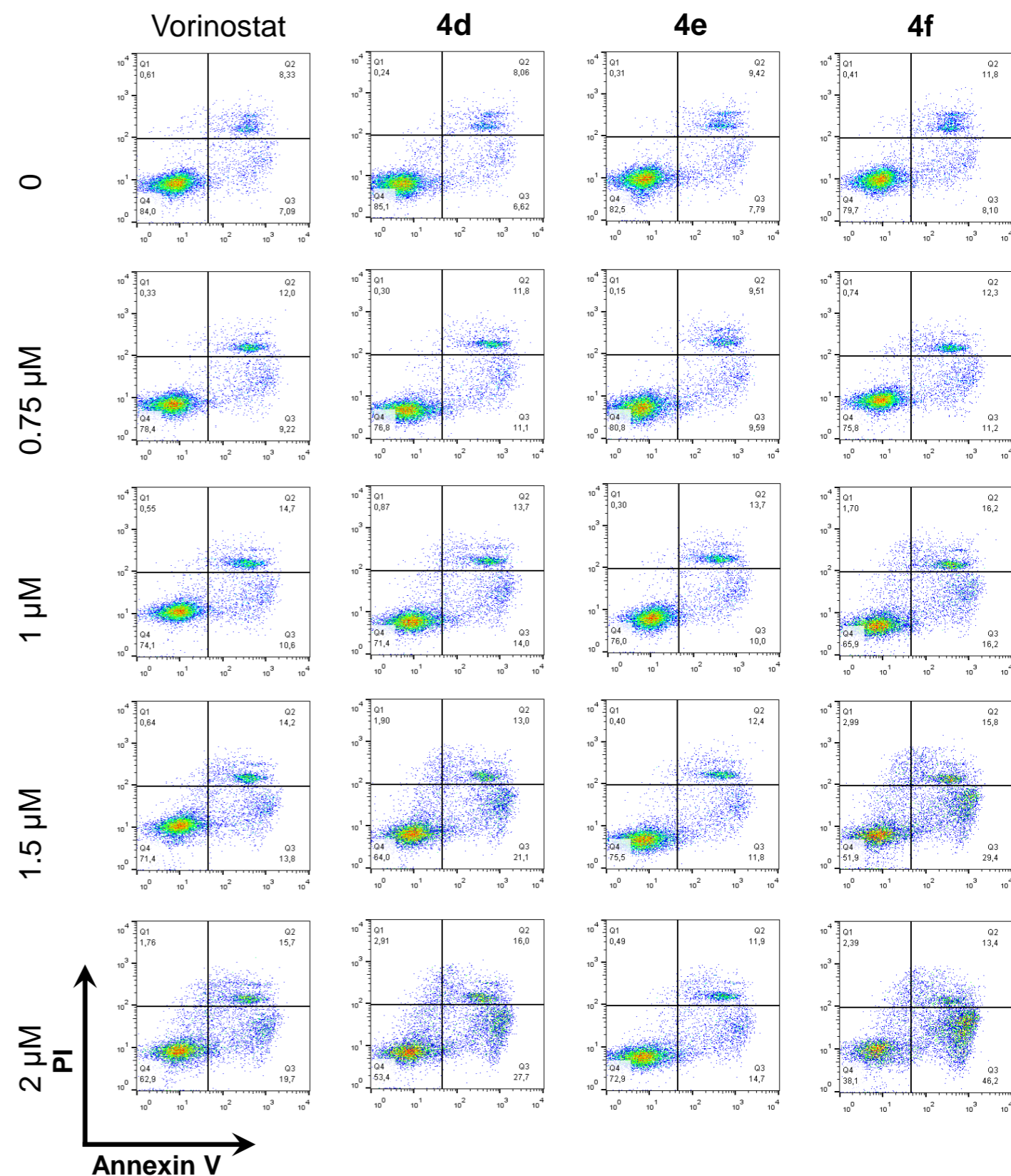

Supplement: Supplementary file 4 — Supplementary Figure 2 [file 41420_2025_2446_MOESM4_ESM.pdf]

**A****NB4-R2 cells**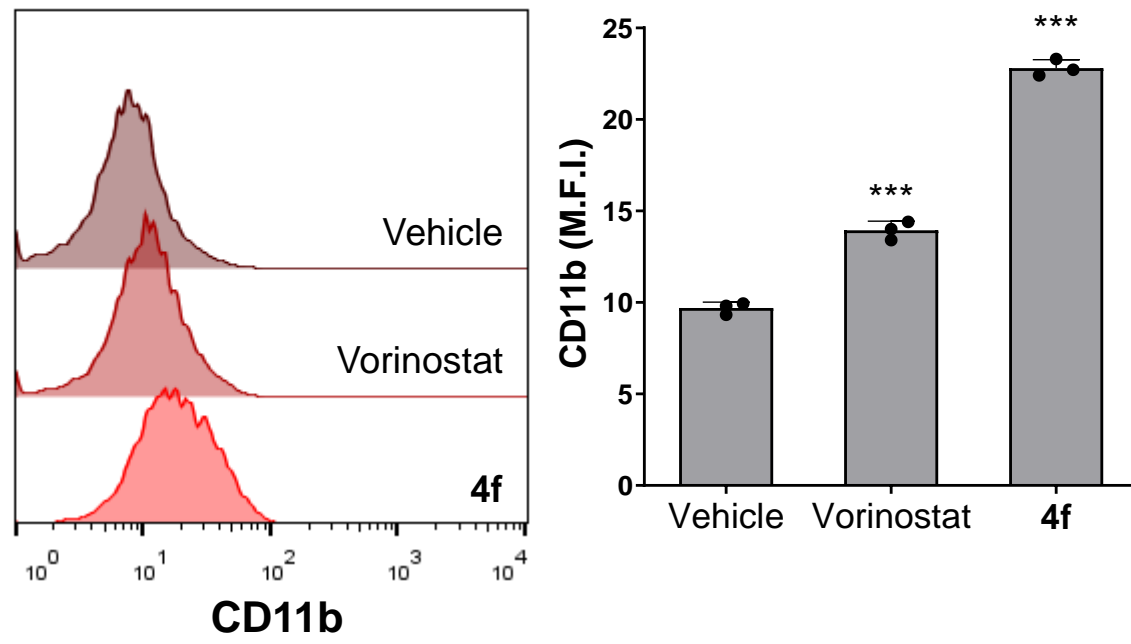**THP-1 cells**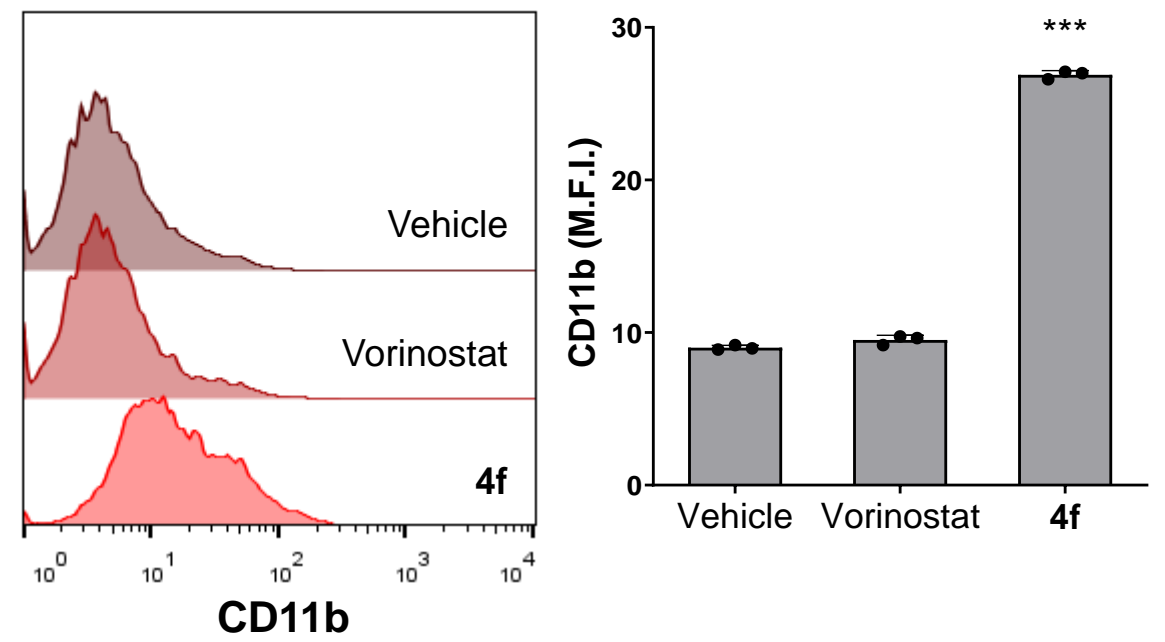**B****Vehicle****Vorinostat****4f****NB4-R2 cells**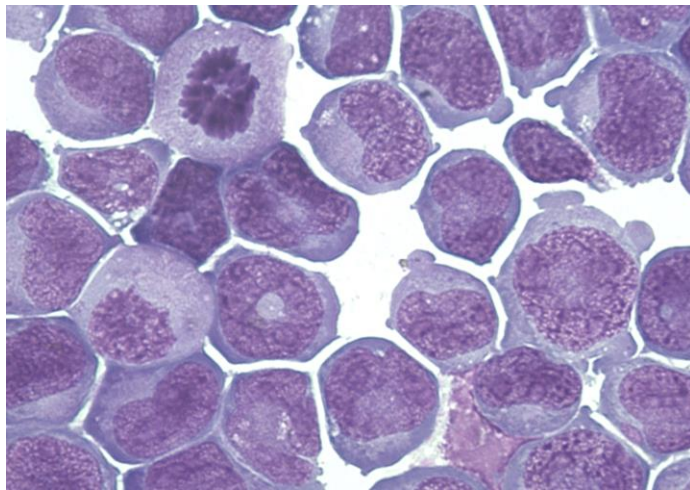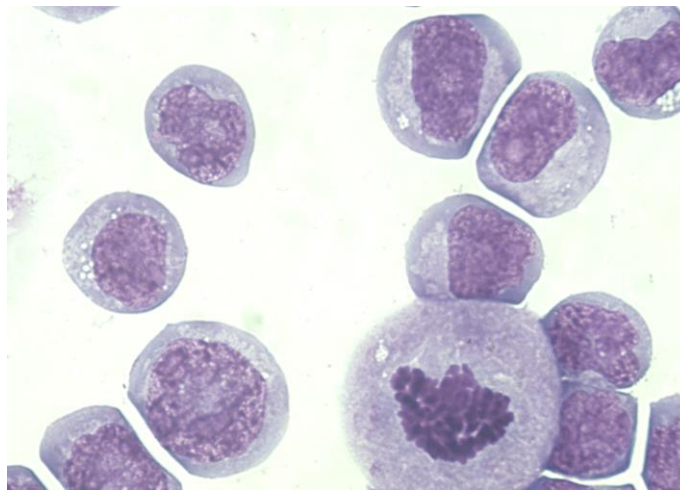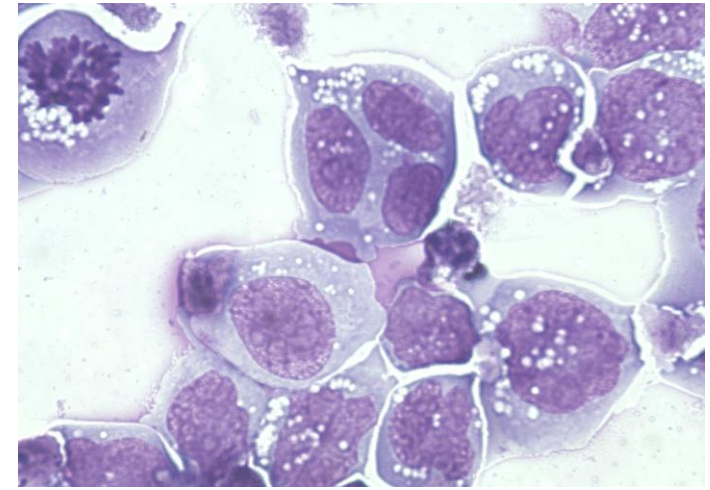**THP-1 cells**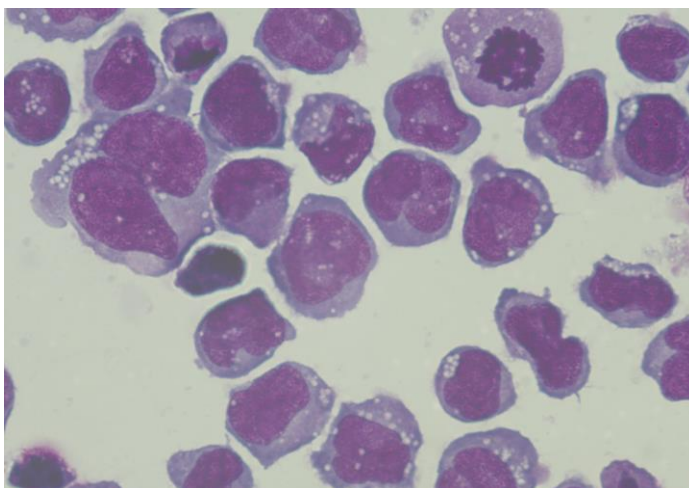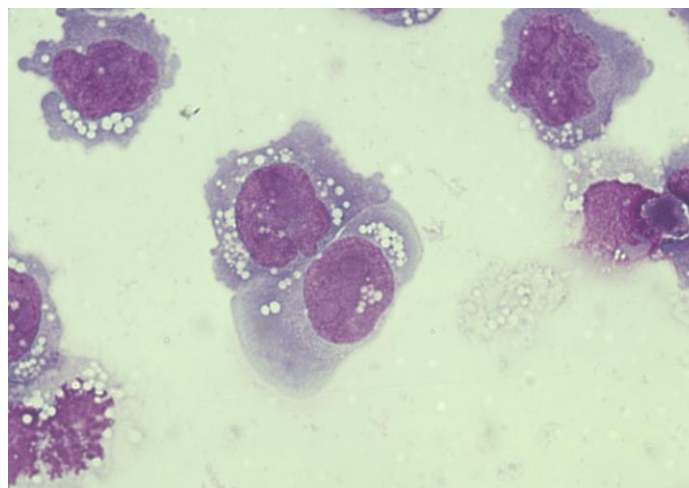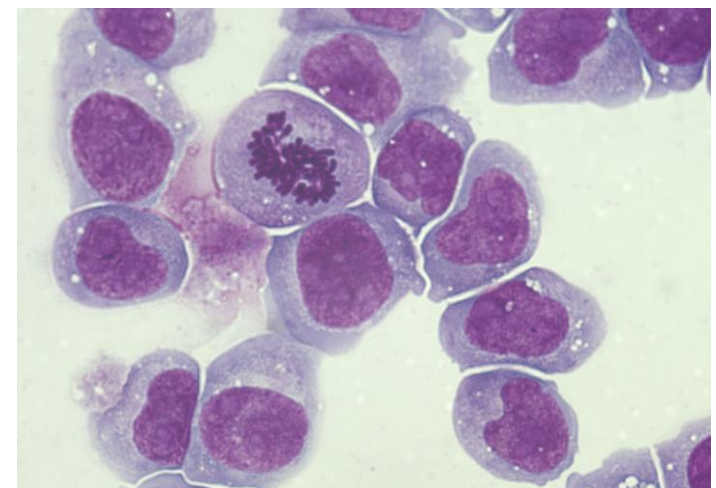

Supplement: Supplementary file 5 — Supplementary Figure 3 [file 41420_2025_2446_MOESM5_ESM.pdf]

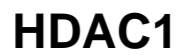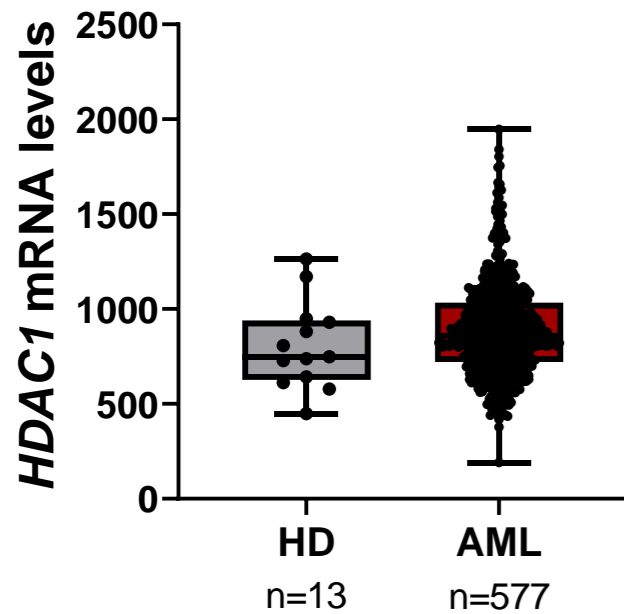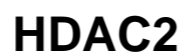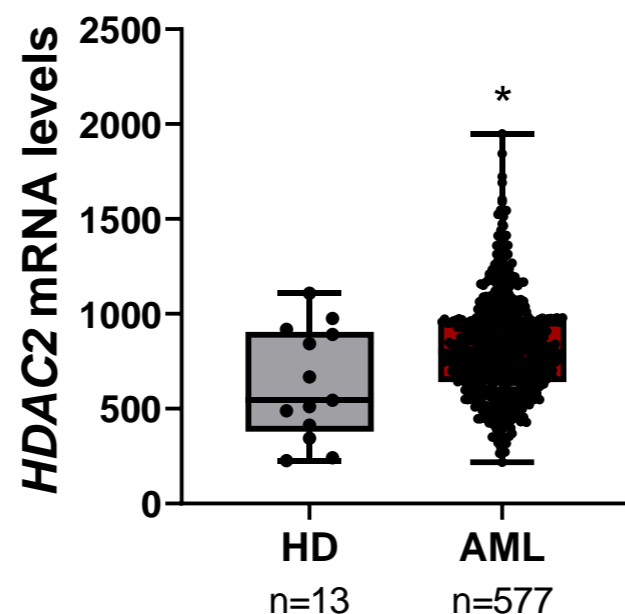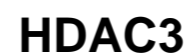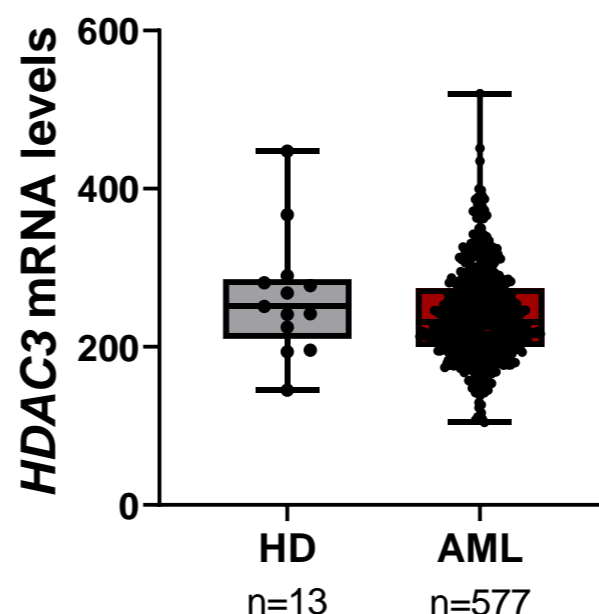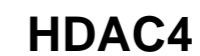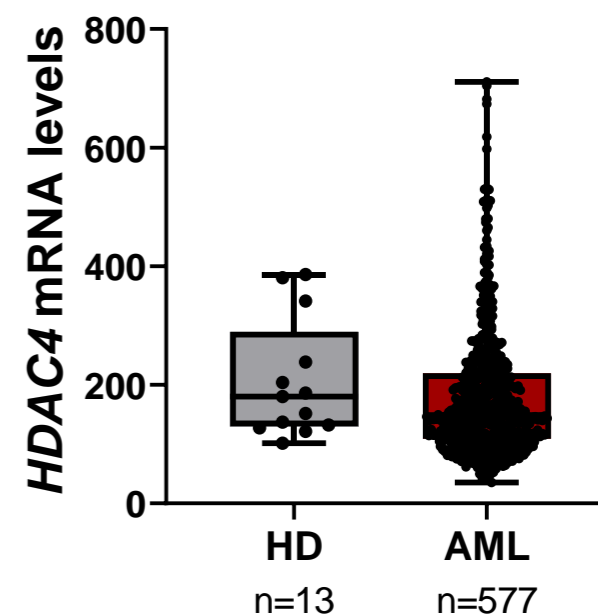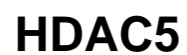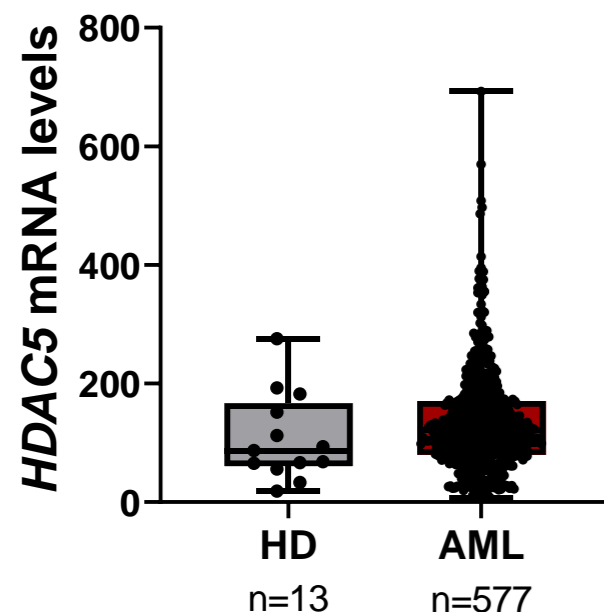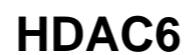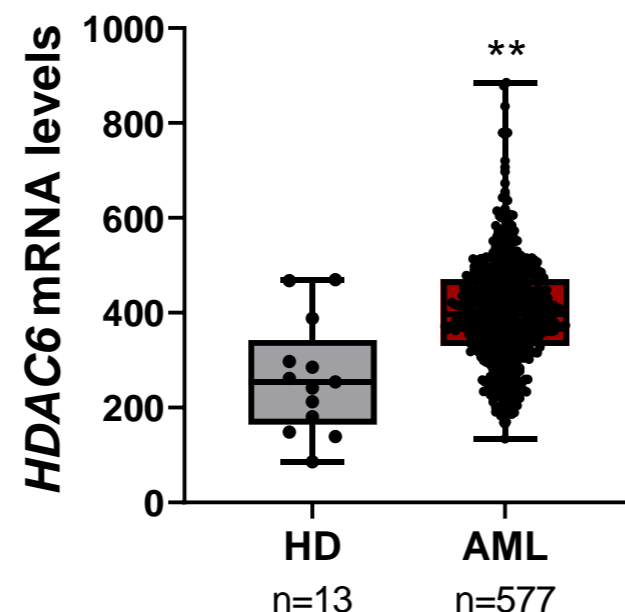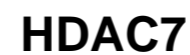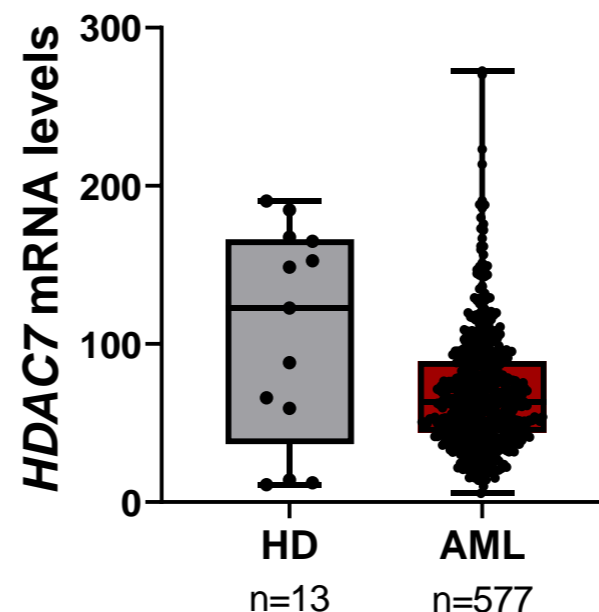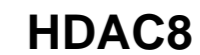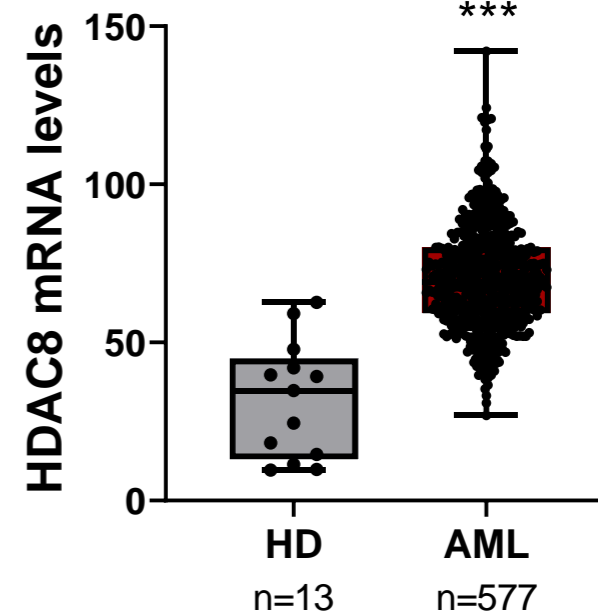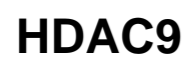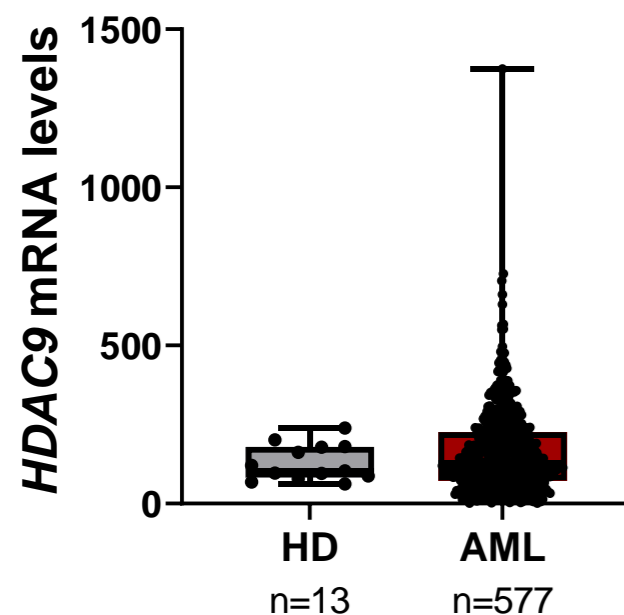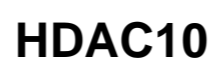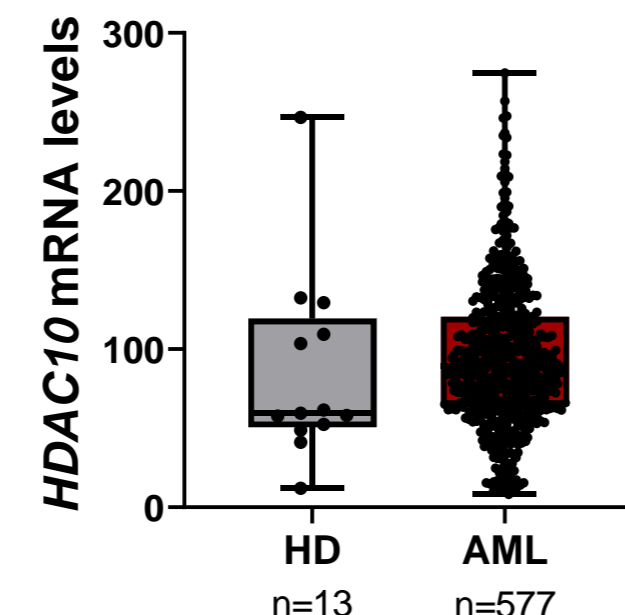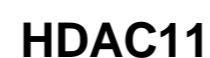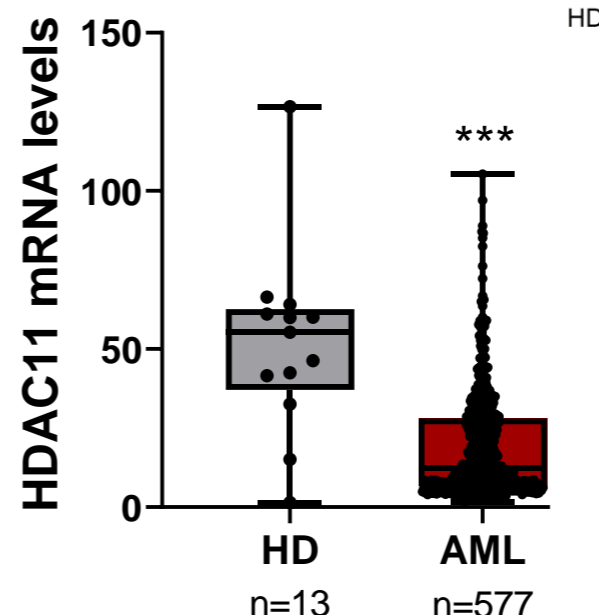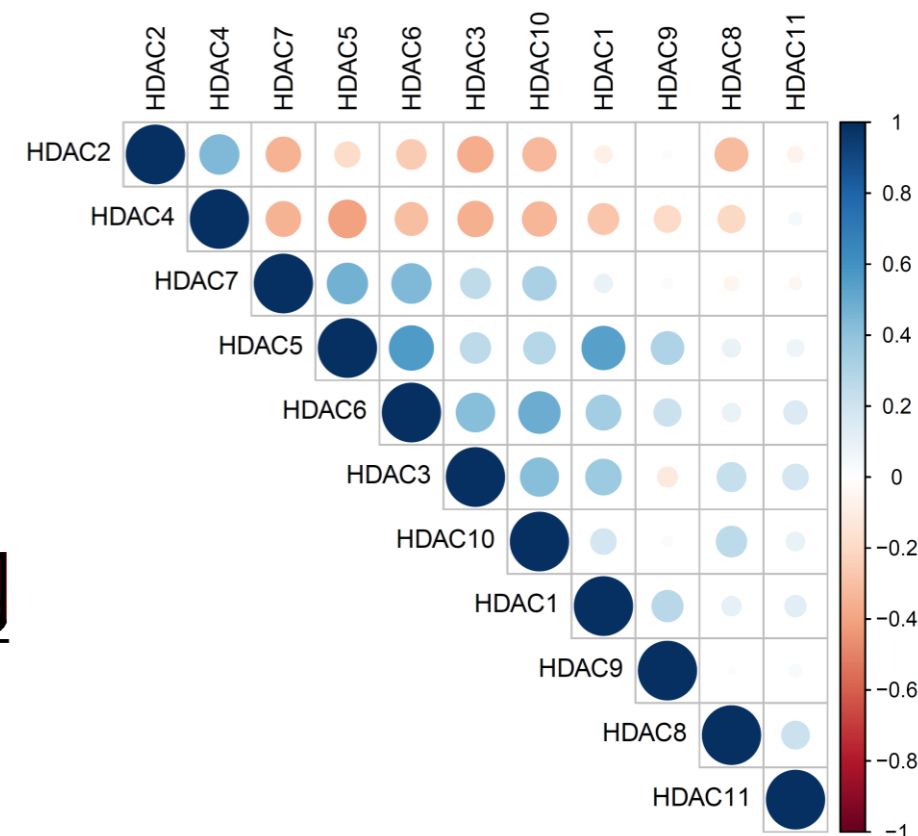

Supplement: Supplementary file 6 — Supplementary Figure 4 [file 41420_2025_2446_MOESM6_ESM.pdf]

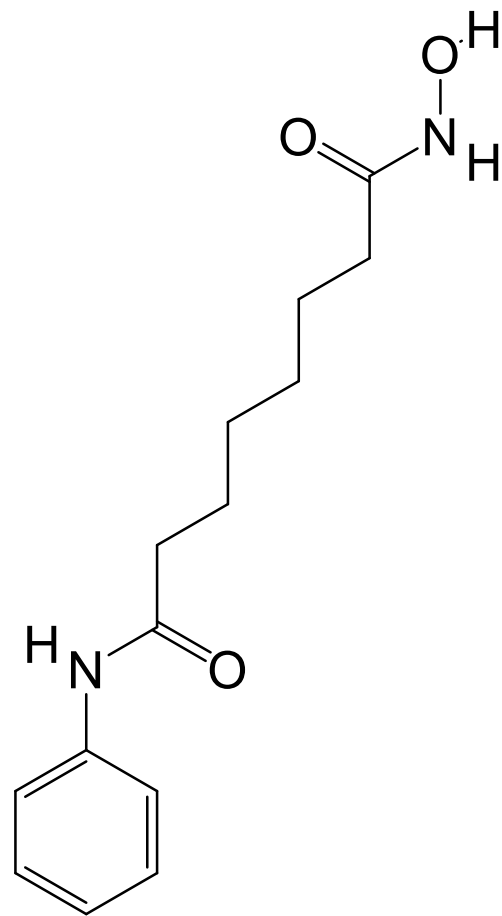

**Vorinostat**

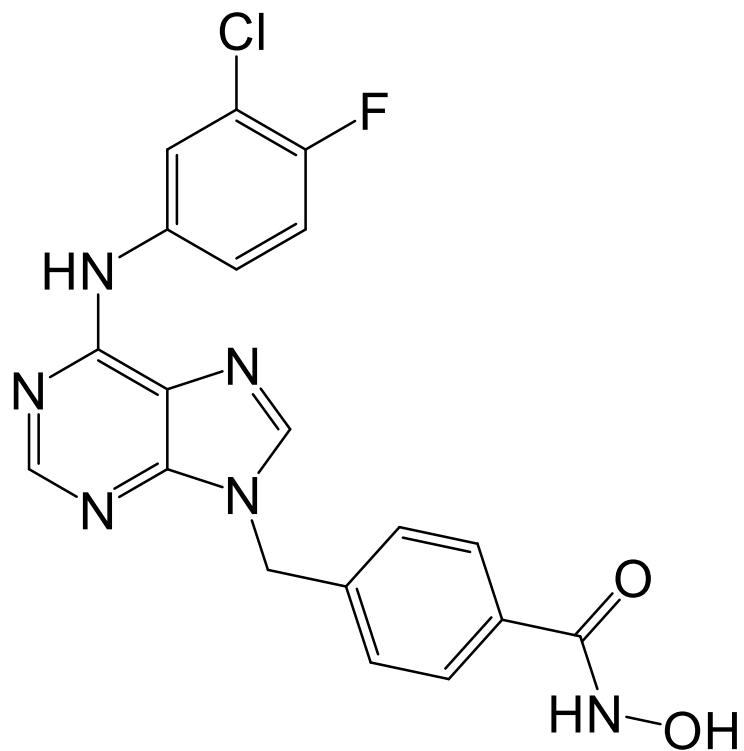

**4d**

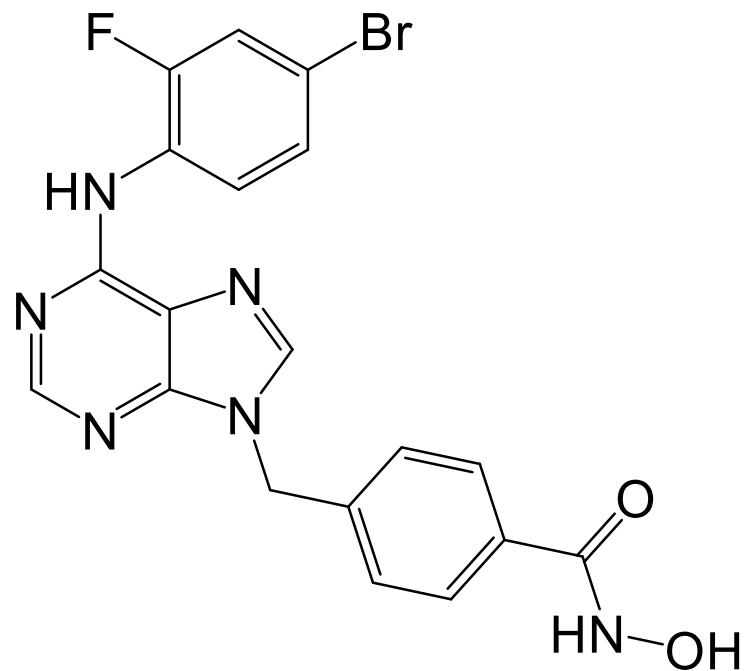

**4e**

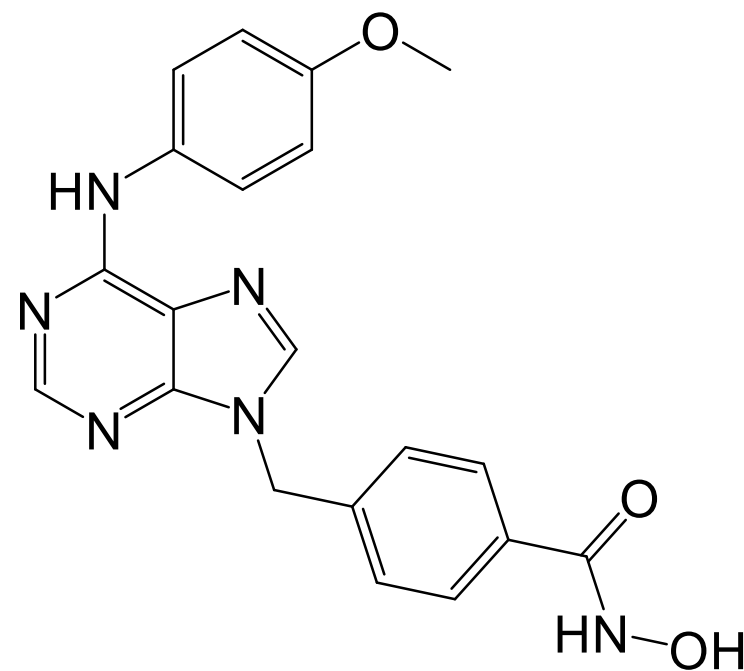

**4f**

Supplement: Supplementary file 9 — Supplementary Figure 7 [file 41420_2025_2446_MOESM9_ESM.pdf]

Whole blots ref. Figure 5

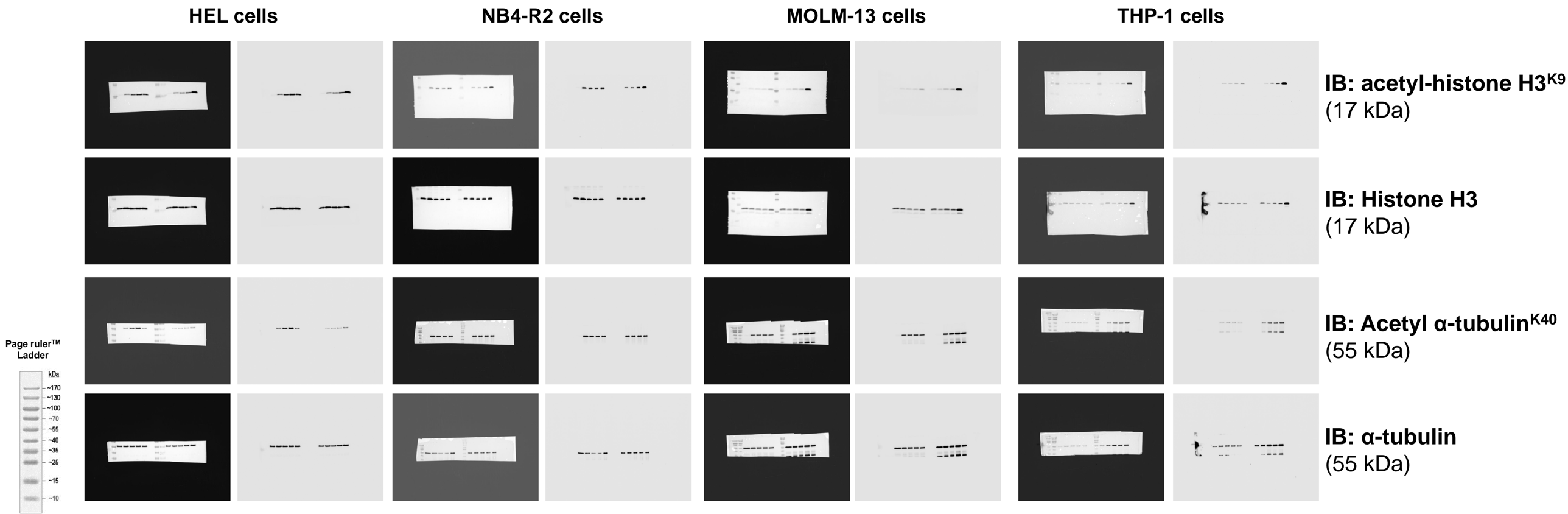

Whole blots ref. Supplementary Figure 5

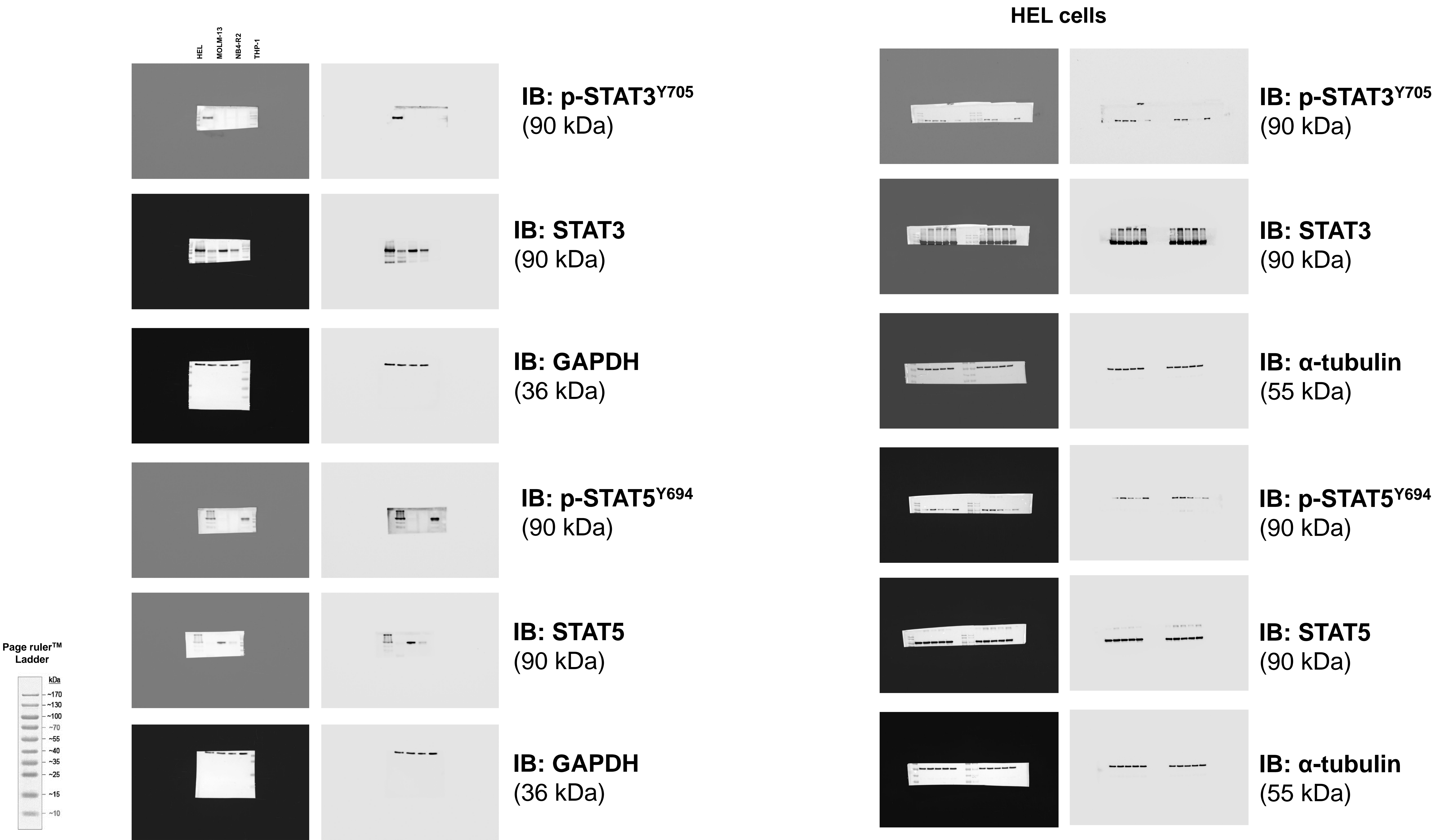

Supplement: Supplementary file 10 — Supplementary Figure 8 [file 41420_2025_2446_MOESM10_ESM.pdf]
