# Supplementary material for: Enhancing venetoclax efficacy in leukemia through association with HDAC inhibitors
Source: Cell Death Discov. 2025 Apr 6;11:147. doi: 10.1038/s41420-025-02446-4 (PMC11972356; doi:10.1038/s41420-025-02446-4)
Supplement: Supplementary file 7 — Supplementary Figure 5 [file 41420_2025_2446_MOESM7_ESM.pdf]

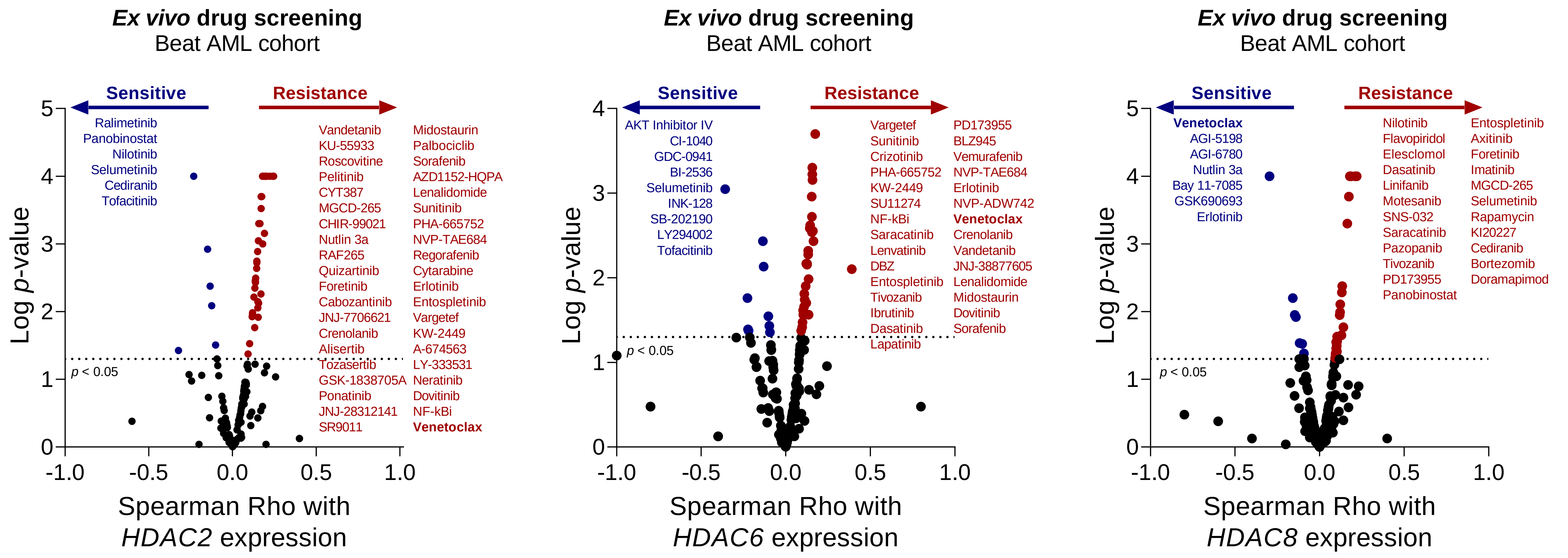

Ex vivo drug screening
Beat AML cohort
Sensitive
Resistance
Ralimetinib
Panobinostat
Nilotinib
Selumetinib
Cediranib
Tofacitinib
Vandetanib
KU-55933
Roscovitine
Pelitinib
CYT387
MGCD-265
CHIR-99021
Nutlin 3a
RAF265
Quizartinib
Foretinib
Cabozantinib
JNJ-7706621
Crenolanib
Alisertib
Tozasertib
GSK-1838705A
Ponatinib
JNJ-28312141
SR9011
Midostaurin
Palbociclib
Sorafenib
AZD1152-HQPA
Lenalidomide
Sunitinib
PHA-665752
NVP-TAE684
Regorafenib
Cytarabine
Erlotinib
Entospletinib
Vargetef
KW-2449
A-674563
LY-333531
Neratinib
Dovitinib
NF-kBi
Venetoclax
p < 0.05
Log p-value
Spearman Rho with
HDAC2 expression
Ex vivo drug screening
Beat AML cohort
Sensitive
Resistance
AKT Inhibitor IV
CI-1040
GDC-0941
BI-2536
Selumetinib
INK-128
SB-202190
LY294002
Tofacitinib
Vargetef
Sunitinib
Crizotinib
PHA-665752
KW-2449
SU11274
NF-kBi
Saracatinib
Lenvatinib
DBZ
Entospletinib
Tivozanib
Ibrutinib
Dasatinib
Lapatinib
PD173955
BLZ945
Vemurafenib
NVP-TAE684
Erlotinib
NVP-ADW742
Venetoclax
Crenolanib
Vandetanib
JNJ-38877605
Lenalidomide
Midostaurin
Dovitinib
Sorafenib
p < 0.05
Log p-value
Spearman Rho with
HDAC6 expression
Ex vivo drug screening
Beat AML cohort
Sensitive
Resistance
Venetoclax
AGI-5198
AGI-6780
Nutlin 3a
Bay 11-7085
GSK690693
Erlotinib
Nilotinib
Flavopiridol
Elesclomol
Dasatinib
Linifanib
Motesanib
SNS-032
Saracatinib
Pazopanib
Tivozanib
PD173955
Panobinostat
Entospletinib
Axitinib
Foretinib
Imatinib
MGCD-265
Selumetinib
Rapamycin
KI20227
Cediranib
Bortezomib
Doramapimod
p < 0.05
Log p-value
Spearman Rho with
HDAC8 expression
