# Supplementary material for: Enhancing venetoclax efficacy in leukemia through association with HDAC inhibitors
Source: Cell Death Discov. 2025 Apr 6;11:147. doi: 10.1038/s41420-025-02446-4 (PMC11972356; doi:10.1038/s41420-025-02446-4)
Supplement: Supplementary file 8 — Supplementary Figure 6 [file 41420_2025_2446_MOESM8_ESM.pdf]

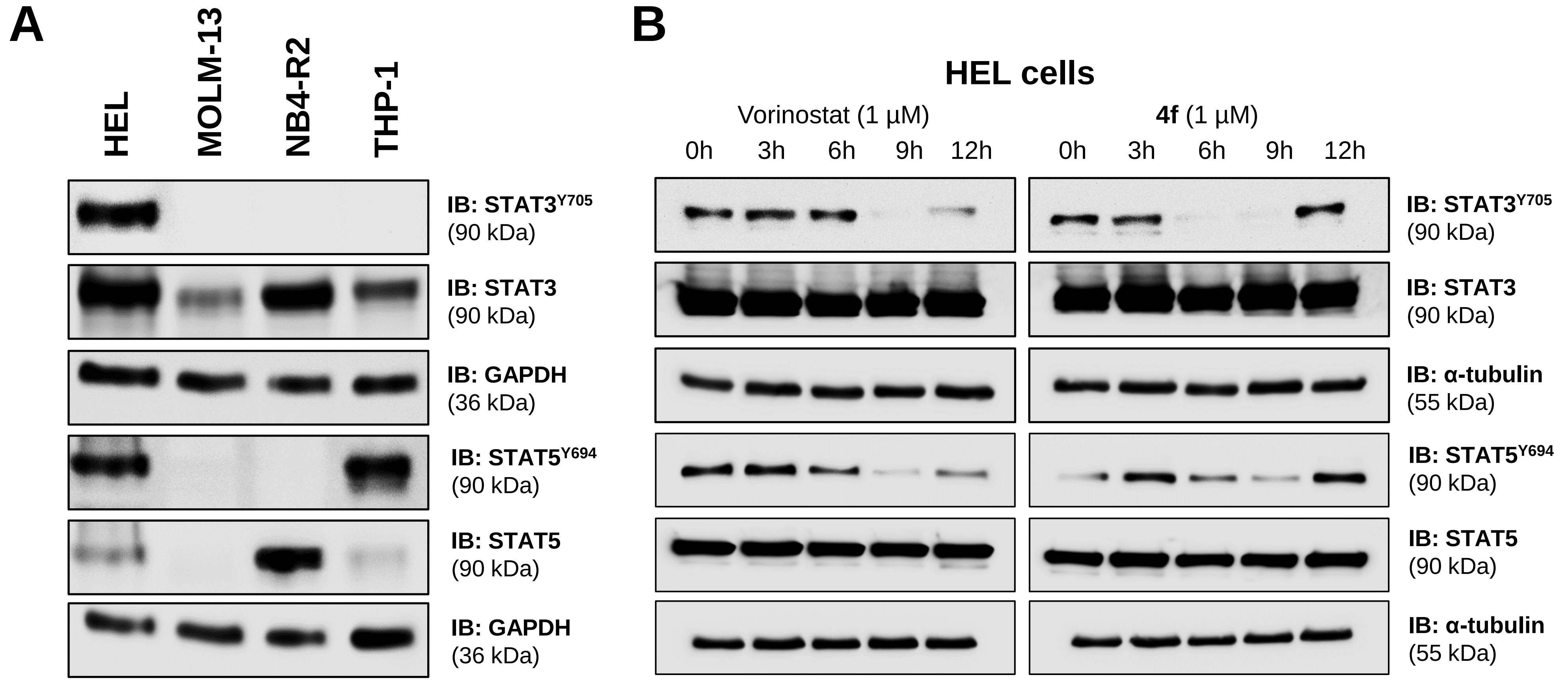
A
HEL
MOLM-13
NB4-R2
THP-1
IB: STAT3Y705
(90 kDa)
IB: STAT3
(90 kDa)
IB: GAPDH
(36 kDa)
IB: STAT5Y694
(90 kDa)
IB: STAT5
(90 kDa)
IB: GAPDH
(36 kDa)
B
HEL cells
Vorinostat (1 µM)
4f (1 µM)
0h
3h
6h
9h
12h
0h
3h
6h
9h
12h
IB: STAT3Y705
(90 kDa)
IB: STAT3
(90 kDa)
IB: α-tubulin
(55 kDa)
IB: STAT5Y694
(90 kDa)
IB: STAT5
(90 kDa)
IB: α-tubulin
(55 kDa)
